# Mosquito Microbiome Dynamics, a Background for Prevalence and Seasonality of West Nile Virus

**DOI:** 10.3389/fmicb.2017.00526

**Published:** 2017-04-04

**Authors:** Eva Novakova, Douglas C. Woodhams, Sonia M. Rodríguez-Ruano, Robert M. Brucker, Jonathan W. Leff, Amin Maharaj, Amnon Amir, Rob Knight, James Scott

**Affiliations:** ^1^Faculty of Science, University of South BohemiaCeske Budejovice, Czechia; ^2^Biology Centre of ASCR, Institute of ParasitologyCeske Budejovice, Czechia; ^3^Department of Biology, University of Massachusetts BostonBoston, MA, USA; ^4^Rowland Institute, Harvard UniversityCambridge, MA, USA; ^5^Cooperative Institute for Research in Environmental Sciences, University of ColoradoBoulder, CO, USA; ^6^Department of Ecology and Evolutionary Biology, University of ColoradoBoulder, CO, USA; ^7^Sporometrics IncToronto, ON, Canada; ^8^Department of Computer Science and Engineering, Center for Microbiome Innovation, University of California San DiegoLa Jolla, CA, USA; ^9^Department of Pediatrics, University of California San DiegoLa Jolla, CA, USA; ^10^Division of Occupational and Environmental Health, Dalla Lana School of Public Health, University of TorontoToronto, ON, Canada

**Keywords:** *Aedes vexans*, *Wolbachia*, *Culex pipiens*, arbovirus, flaviviridae, disease ecology

## Abstract

Symbiotic microbial communities augment host phenotype, including defense against pathogen carriage and infection. We sampled the microbial communities in 11 adult mosquito host species from six regions in southern Ontario, Canada over 3 years. Of the factors examined, we found that mosquito species was the largest driver of the microbiota, with remarkable phylosymbiosis between host and microbiota. Seasonal shifts of the microbiome were consistently repeated over the 3-year period, while region had little impact. Both host species and seasonal shifts in microbiota were associated with patterns of West Nile virus (WNV) in these mosquitoes. The highest prevalence of WNV, with a seasonal spike each year in August, was in the *Culex pipiens/restuans* complex, and high WNV prevalence followed a decrease in relative abundance of *Wolbachia* in this species. Indeed, mean temperature, but not precipitation, was significantly correlated with *Wolbachia* abundance. This suggests that at higher temperatures *Wolbachia* abundance is reduced leading to greater susceptibility to WNV in the subsequent generation of *C. pipiens/restuans* hosts. Different mosquito genera harbored significantly different bacterial communities, and presence or abundance of *Wolbachia* was primarily associated with these differences. We identified several operational taxonomic units (OTUs) of *Wolbachia* that drive overall microbial community differentiation among mosquito taxa, locations and timepoints. Distinct *Wolbachia* OTUs were consistently found to dominate microbiomes of *Cx. pipiens/restuans*, and of *Coquilletidia perturbans*. Seasonal fluctuations of several other microbial taxa included *Bacillus cereus, Enterococcus, Methylobacterium, Asaia, Pantoea, Acinetobacter johnsonii, Pseudomonas*, and *Mycoplasma*. This suggests that microbiota may explain some of the variation in vector competence previously attributed to local environmental processes, especially because *Wolbachia* is known to affect carriage of viral pathogens.

## Introduction

Metazoa harbor diverse microbial communities (microbiota) largely dominated by bacteria (Bordenstein and Theis, 2015; Yadav et al., 2015). The microbiota modifies the ability of a host to be affected by, and to transmit, pathogens. Thus, understanding the relationship between microbiota and arthropod disease vectors, including mosquitoes, may impact mitigation of emerging infectious diseases (Dennison et al., 2014; Van Treuren et al., 2015).

Recently emerging vector-borne diseases have been linked to the introduction of non-native insect vectors and to changing ecological conditions including climate, urbanization, and greater human intrusion into areas where vectors and pathogens prevail (Bonizzoni et al., 2013). However, it is not known whether vector competence (i.e., the ability to transmit pathogens) is shaped mainly by environmental conditions, genetic background of the insect vector, or by the vector microbiota. Environmental factors and vector genotype both affect insect body size (Alto et al., 2008) and immunity status (Murdock et al., 2013), two traits that affect pathogen transmission. The microbiota may also influence disease dynamics.

Recent studies indicate that each mosquito species harbors specific microbiota even when larvae are raised under common conditions (Coon et al., 2014; Brooks et al., 2016). This distinction holds even when host species share habitat and are closely related and morphologically indistinct (Muturi et al., 2016). However, environmental conditions can also influence the microbiota of insect disease vectors (e.g., Jones et al., 2010; Tchioffo et al., 2016). It remains to be clarified whether mosquito genotype, region, or season is dominant in structuring microbial communities. For example, do differences in bacterial communities among mosquito species depend on season? Are there specific bacteria important in structuring the microbiota that dependent on regional environmental acquisition?

Some microbes, in particular the vertically transmitted endosymbiotic bacteria *Wolbachia*, have been shown to modulate pathogen infection and transmission in insects (e.g., Dennison et al., 2014; Dutra et al., 2016). *Wolbachia* endosymbionts affect the capacity of mosquitoes to carry specific parasites and viral pathogens (Martinez et al., 2014). *Wolbachia*-mediated effects in different hosts and RNA viruses range from reduced virus proliferation and transmission (Lu et al., 2012) to enhanced infection rates (Dodson et al., 2014). For instance, dengue virus can be suppressed by *Wolbachia* strains transinfected in *Aedes aegypti* (Hoffmann et al., 2011; Sinkins, 2013), and, at sufficiently high densities, in *Aedes albopictus* (Lu et al., 2012; Bian et al., 2013). In contrast, *Wolbachia* enhances WNV replication in *Ae. aegypti* cell line but inhibits virus assembly (Hussain et al., 2013), showing that *Wolbachia* protective phenotypes can rely on several distinct mechanisms. These mechanisms include resource competition (e.g., Moreira et al., 2009), immune stimulation (e.g., Pan et al., 2012), and small noncoding RNAs produced by *Wolbachia* that can regulate host genes (Mayoral et al., 2014). The protective effect of *Wolbachia* against Flaviviruses including Dengue and Zika (Dutra et al., 2016) has even been deployed deliberately for vector control. Artificially *Wolbachia*-infected mosquitoes were released in virus-endemic zones to spread the infection-reducing *Wolbachia* through the mosquito population (e.g., http://www.eliminatedengue.com).

Although artificially introduced *Wolbachia* strains can confer antiviral protection to new mosquito hosts (Bourtzis et al., 2014), similar effects have seldom been shown for native *Wolbachia* infections. For instance, while native *Wolbachia* infection in *Culex quinquefasciatus* inhibits dissemination and transmission of West Nile virus (WNV), the resistance is modest compared to the effects of *Wolbachia* in *Drosophila melanogaster* (Glaser and Meola, 2010). Natural resistance to WNV in field sampled *Cx. quinquefasciatus* and *Cx. pipiens* depends on sufficiently high *Wolbachia* densities, and is likely limited to specific populations (Glaser and Meola, 2010). In contrast to protection conferred by introduced *Wolbachia* strains, co-evolution of *Cx. pipiens* with natural *Wolbachia* infection favored vector competence and transmission of *Plasmodium relictum* (Zélé et al., 2014). *Wolbachia* may increase mosquito longevity and protect against *Plasmodium*-induced mortality (Zélé et al., 2014).

*Wolbachia* symbionts, though of unquestionable importance, are just one constituent of the entire mosquito-associated microbiota. Arguably, intracellular bacteria may not be considered part of the microbiota as they may have limited interactions with microbial communities in the mouth, gut, skin, or other organs. Because the gut epithelial cells are the initial site of viral proliferation, gut microbiota may play a crucial role in antiviral resistance and vector competence of mosquito species or populations (Moreira et al., 2009). One field of thought is that rather than stemming from co-evolution, the microbiota in mosquitoes might represent opportunistic environmental colonization (Osei-Poku et al., 2012). Undefined local processes were found to underlie spatial and temporal variation in vector competence for WNV in *Cx. pipiens* and *Cx. restuans* (Kilpatrick et al., 2010), and these results might be explained by location-specific environmentally acquired microbes.

To resolve these issues, we examined the microbiota, including *Wolbachia* relative abundance, in respect to host taxa, seasonality and WNV infection status in natural populations of 11 mosquito species in Ontario, Canada. Specifically, we tested whether the dominant drivers of microbial community variation were host species, geography, or season. We also tested whether any of the variation correlated with WNV infection, providing insight into possible effects on vector competence.

## Materials and methods

### Sample origin, RNA, and DNA extraction

**Figure 1:**
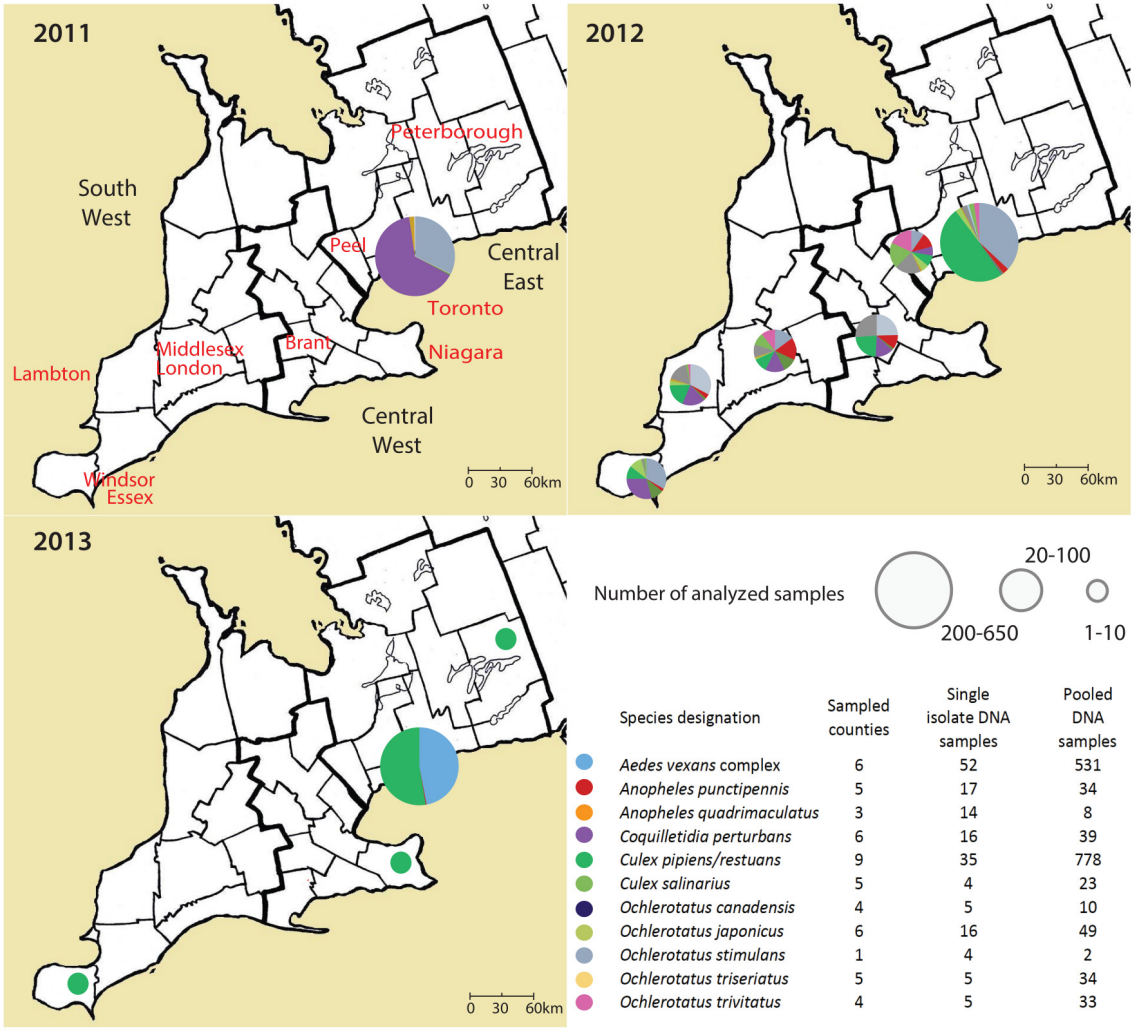
**An overview on analyzed samples, their geographical background and year of sampling in Ontario, Canada**. Single mosquito samples and pooled mosquito samples were analyzed separately.

Adult female mosquitos of 11 species were collected between 2011 and 2013 from Toronto, and 9 different geographical regions in Ontario, Canada (Table 1, Figure 1). Traps were set at residential properties, and at municipal buildings or parks. Details of sampling design and methods are available in Supplemental Materials (Table S1, Figure S1). The collected insects were frozen, identified morphologically to species, and pooled from each trap into samples containing 1–50 mosquitoes of the same species (Table 1). If only one individual of a species was present in a trap, this provided an unpooled sample with only one mosquito.

**Table 1:** **Mosquito species captured and sampled for West Nile virus (WNV) from 2011 to 2013 in Ontario, Canada**.

**Species**	**N samples**	**Mean N mosquitoes/sample**	**N samples tested for WNV**	**WNV+ samples**	**WNV prevalence (%)**
*Aedes vexans complex*	2,947	14.3	1,868	15	0.8
*Ochlerotatus canadensis*	201	4.1	19	0	0.0
*Ochlerotatus japonicus*	1,049	5.5	1,037	1	0.1
*Ochlerotatus stimulans*	143	9.3	143	0	0.0
*Ochlerotatus triseriatus*	459	4.1	458	1	0.2
*Ochlerotatus trivitatus*	472	8.2	472	0	0.0
*Coquilletidia perturbans*	1,139	16.3	1	0	0.0
*Culex pipiens/restuans*	3,652	12.2	3,648	297	8.1
*Anopheles punctipennis*	406	2.4	406	1	0.2
*Anopheles quadrimaculatus*	70	2.3	69	0	0.0
*Culex salinarius*	109	3.4	109	4	3.7
*Culex tarsalis*	2	2.5	2	0	0.0
Total	10,649	11.3	8,232	319	3.9

Following homogenization and centrifugation, RNA was extracted from 200 μL of supernatant (Supplemental Methods). RNA from pools found to be positive by the WN3′ NC primer-probe combination was re-tested using the WNENV primer-probe combination to confirm WNV positivity. The Ontario Ministry of Health mandated that this protocol be utilized in the mosquito surveillance program for the testing of WNV in mosquito pools. The amplicon sizes are 103 bp for the WN3′NC primers and 70 bp for the WNENV primers as previously reported (Lanciotti et al., 2000).

From each sample, DNA was extracted according to the Earth Microbiome Project protocol (http://www.earthmicrobiome.org/emp-standard-protocols/dna-extraction-protocol/) using the MoBio PowerSoil DNA Isolation Kit for 2,298 samples (232 single mosquito isolates and 2,066 pooled samples representing gDNA from 2 to 50 individuals of the same species).

### WNV diagnosis

A total of 8,232 samples were tested for WNV between 2011 and 2013 using a TaqMan real time PCR assay according to Lanciotti et al. (2000; Supplemental Methods and Table S2).

### Data generation and processing

Genomic DNA from 2,298 samples, along with the negative controls, was amplified according to the EMP protocol (http://www.earthmicrobiome.org/protocols-and-standards/16s/) using the 515/806 primer pair and analyzed using barcoded sequencing on 3 lanes of Illumina HiSeq 2000. Raw reads of 125 bp were processed using UPARSE (Edgar, 2013) according to the following scheme: (i) demultiplexing reflecting the raw data barcodes, (ii) quality filtering using the maxee parameter set to 0.5, (iii) dereplicating identical sequences, and (iv) removing singletons to create *de novo* database (v) mapping raw reads to the database to generate sequence counts per OTU and sample. The number of sequences per sample was approximately 50,000 on average, and samples with <1,000 reads were omitted. While the number of the OTUs appeared extremely high in comparison with previous studies (e.g., Coon et al., 2014), the raw data were reanalyzed using Deblur (https://github.com/biocore/deblur; AA, submitted). This algorithm does not inflate the number of OTUs and produced a more realistic picture of the mosquito microbiota. Deblur is a de-noising method which, after removal of PCR and read-error derived reads, can identify sequences with as little as one nucleotide difference over the sequence region, as opposed to clustering based approaches such as UPARSE, which cluster together sequences more similar than a given noise derived threshold (usually 97%). For instance, for the 11 mosquito species analyzed, deblurring revealed 17 *Wolbachia* clusters (called OTUs in a broader sense here after, Figure S3) compared to 148 OTUs generated by UPARSE. Another useful property of Deblur is that it is stable—it is run on each sample independently and the same sequence in different samples will be identified as the same OTU. Whereas, in de-novo clustering methods, all samples need to be processed together since otherwise the same sequence can be assigned to two different OTUs, depending on the neighboring sequences.

After sequence processing, negative controls were checked for contaminants. Two out of six negative controls were clean (<30 sequences), while the other four showed some amplification (above 5,000 sequences). Particularly, most of the sequences in those negative controls corresponded to two OTUs, identified as Enterobacteriaceae (54% of the reads in one control) and Pseudomonadaceae (between 43 and 63% of the reads in three controls). The Pseudomonadaceae OTU showed high frequency (90%) and mean abundance (269.4 ± 230.7 sequences, normalized at 1,000 sequences per sample) in our samples. This result agrees with previous work that found this family of bacteria in different mosquito species (Minard et al., 2013). Accordingly, the amplification in these controls could be caused by cross-contamination from our samples during processing, more likely than from an external source. On the other hand, the Enterobacteriaceae OTU showed low frequency (20%) and mean abundance (19 ± 70.2 sequences per sample, normalized at 1,000 sequences) in our samples, suggesting that it could be a real contaminant. However, the presence of insect-specific symbionts such as *Wolbachia* in high abundance in our samples (but not in the negative controls), and its mosquito species-specific pattern, suggest that there was no significant effect of any contamination in our samples that would affect further analyses.

Clustered OTUs consisted of sequences matching bacterial and mitochondrial 16S rRNA genes as well as 18S rRNA gene sequences that were also amplified (presumably because of low primer specificity and low complexity of the analyzed microbiota against an excess of host DNA). 16S rRNA OTUs for analyses of microbiota were retrieved from the complete data set using BlastN searches against 16S rRNA gene sequence database (NCBI). The taxonomic assignment of these OTUs was based on the RDP classifier and Greengenes reference using 97% similarity (Wang et al., 2007). Considering recent findings that 16S rRNA amplicon sequencing can reveal relative quantitative changes in abundance of taxa among samples (D'Amore et al., 2016), the relative abundance of *Wolbachia* in the single isolates was calculated as the percentage of all the 16S rRNA amplicon reads. Other sequences (18 and 16S mitochondrial, plastid and archaeal OTUs) were identified using BlastN (Camacho et al., 2008). This approach enabled a strict quality check (discarding possible contaminants and taxonomically mis-assigned samples using 18S rRNA gene sequences described below).

Although the mosquito specimen identification was solely based on morphology, we took advantage of 18S rRNA amplicons and used those as a molecular marker. Indeed, we retrieved on average 1,118 and 4,376 reads of mosquito 18S rRNA per each of individual and pooled samples, respectively. The data were used as a quality check with the potential to reveal and resolve several methodological artifacts. In particular, artifacts could include incorrect taxonomic assignment based on morphology, species complexes that cannot easily be resolved, and sample contamination from other mosquitoes in the same trap. Clustering of 18S rRNA host sequences (detected here with the universal 16S primers) displayed a clear pattern reflecting the sample taxonomy and allowed for molecular based taxonomic determination on different taxonomic levels. While we could not distinguish between closely related species of *Aedes vexans* complex, or among four *Ochlerotatus* species (*O. canadiensis, O. stimulans, O. triseriatus*, and *O. trivitatus*, all clustered into a single OTU), *O. japonicus* sequences formed another OTU. *Anopheles* species, *An. punctipenis* and *An. quadrimaculatus*, split into two different OTUs with 98% simillarity. Two *Culex* species, *Cx. pipiens/restuans* and *Cx. salinarius* however clustered together into a single OTU. *Coquilletidia perturbans* sequences were represented by a unique cluster.

The following rule was applied to filter out potentially misleading data: Samples with <90% of 18S rDNA sequences in the taxon specific OTU described above, and samples with 0 total reads for host 18S rRNA. These samples were not analyzed within the final dataset. Altogether 102 pooled samples and 21 individual samples were discarded. Taxonomic assignment was corrected for eight samples. Altogether, a subset of 173 single-mosquito samples, and 1,541 pooled samples (2–50 mosquitoes of the same species trapped together) passed the quality control and was further analyzed (Figure 1). The raw sequence data are available at European Bioinformatics Institute database under accession number ERP021438. The dataset is also available at https://qiita.ucsd.edu/ (ID 10815).

### Statistical analyses of the microbial communities

To assess composition and diversity of mosquito associated bacterial communities, two sets of 16S rDNA amplicons were analyzed: single-mosquito samples and pooled samples, following the same workflow. All the analyses were performed in R environment (R Core Team, 2016) using following packages and libraries: datasets, dplyr, stats, biom, vegan, ggplot2, clickme (Wickham, 2009; Oksanen et al., 2013; McMurdie and the biom-format team, 2014; RStudio Team, 2015; Caballero, 2016; R Core Team, 2016). First, the sequencing depth among the samples was normalized by rarifying the data to 1,000 sequences per sample for the single-mosquito samples, and 5,000 sequences per sample for the pooled samples. Normalization of sampling depth is advised for samples ranging widely in sequencing depth (Weiss et al., 2015). Shannon index and richness was used to describe the bacterial diversity among different host species. Kruskal-Wallis tests were used to evaluate differences in diversity among host species. Bray-Curtis dissimilarities calculated from abundance tables were used for further evaluation of selected factors, i.e., host genetic background, geographical background, seasonality (week number), potentially shaping the community profiles. Statistical testing was performed using permutational multivariate ANOVA implemented in R (Adonis function in vegan package; Oksanen et al., 2013). In order to reveal to what extent *Wolbachia* OTUs affect calculated dissimilarities, these OTUs were systematically excluded generating a series of datasets (not shown). The dissimilarities among analyzed microbiomes were then statistically tested as described above for the host species and genus level. Furthermore, a pairwise comparison was performed for each possible species and genera pair for the full dataset and the one missing all the *Wolbachia* OTUs. Constrained ordinations were used to visualize the overall differences among microbiomes of different species and genera. The two control analyses for the exclusion approach were performed eliminating the second most abundant OTU, i.e., *Asaia*, and *Pseudomonas*, the OTU shared by all the mosquito species. All the datasets used in the exclusion analyses underwent rarefaction at the level of 300 reads acceptable for the majority of the samples. QIIME implemented python script *group_significance* was used with Kruskal-Wallis tests to identify bacterial OTUs with significantly different abundances among species.

### Phylosymbiosis analysis

Phylosymbiosis refers to the observation of congruency between host phylogeny and whole microbial community topology, and infers some shared ancestral microbial community. Using the same analysis as presented in Brooks et al. (2016), host phylogenetic trees were constructed using an incomplete multigene matrix of 18S, 28S, COI and NADH available for all analyzed mosquito species in GenBank. The sequences were aligned using Muscle v3.8.31 (Edgar, 2004), and alignments were evaluated using jModelTest v2.1.7 (Darriba et al., 2012). The optimal host tree and bootstrap values were generated in RaxML v8.0.0 (Stamatakis, 2014). The software package ETE 3 (Robinson and Foulds, 1981) was used to determine topological congruencies for the host phylogeny and the beta-diversity of the average community abundancies for each host species. Topographical symmetry and edge similarity for trees was quantified by the normalized Robinson-Foulds (RF) metric (Huerta-Cepas et al., 2016) to determine topological similarity on a scale from 0 (complete congruence) to 1 (incomplete incongruence). Robinson-Foulds metrics were evaluated for Bray-Curtis, unweighted UniFrac, and weighted UniFrac beta-diversity dendrograms at 97% and 99% OTU clustering and compared to a null to determine if the host-microbe congruency is randomly associated (Brooks et al., 2016).

## Results

### Diversity and host species specificity of mosquito microbiota

**Figure 2:**
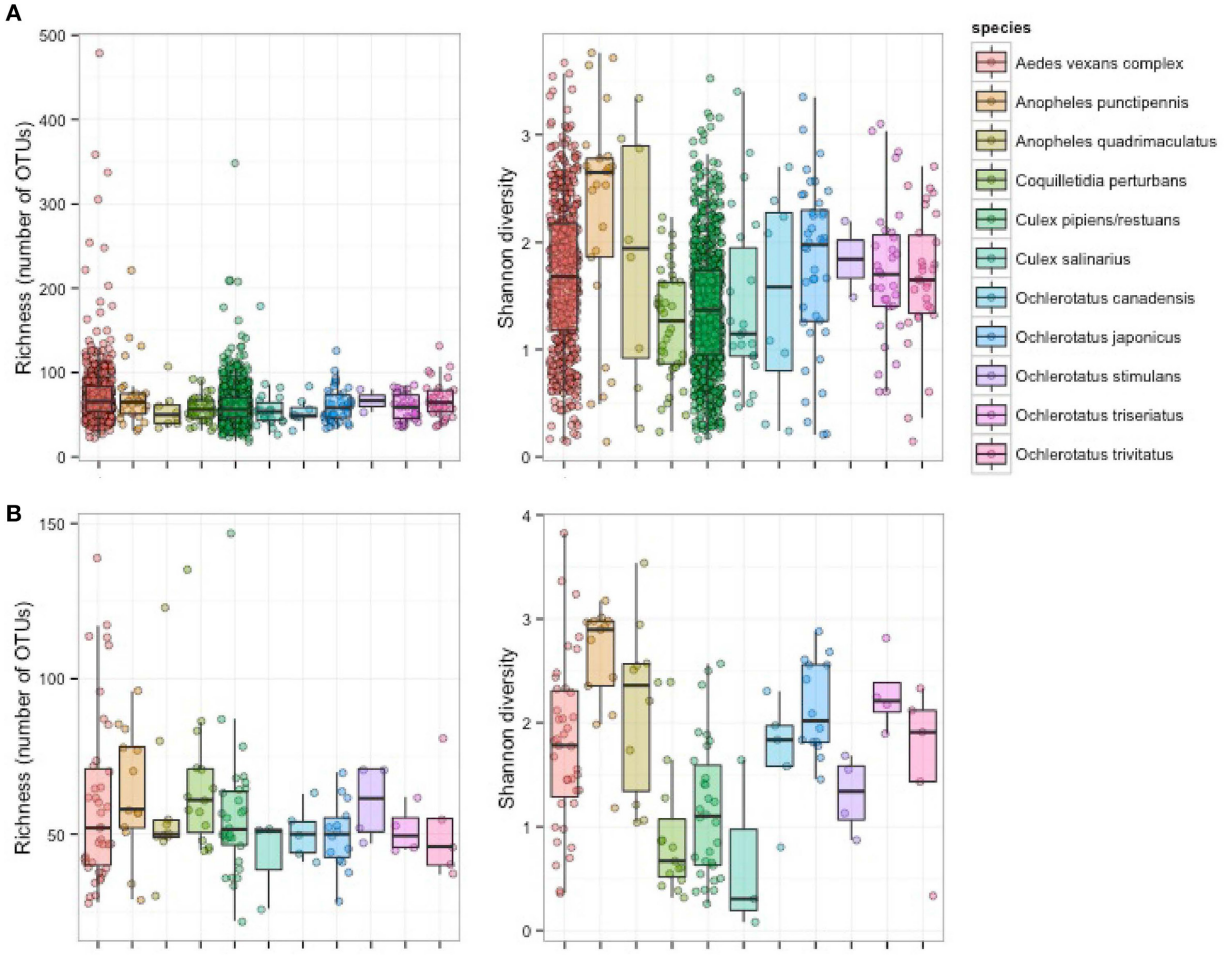
**Richness and alpha diversity of microbiota from 11 mosquito species based on pooled samples (A)** and single mosquito samples **(B)**.

**Figure 3:**
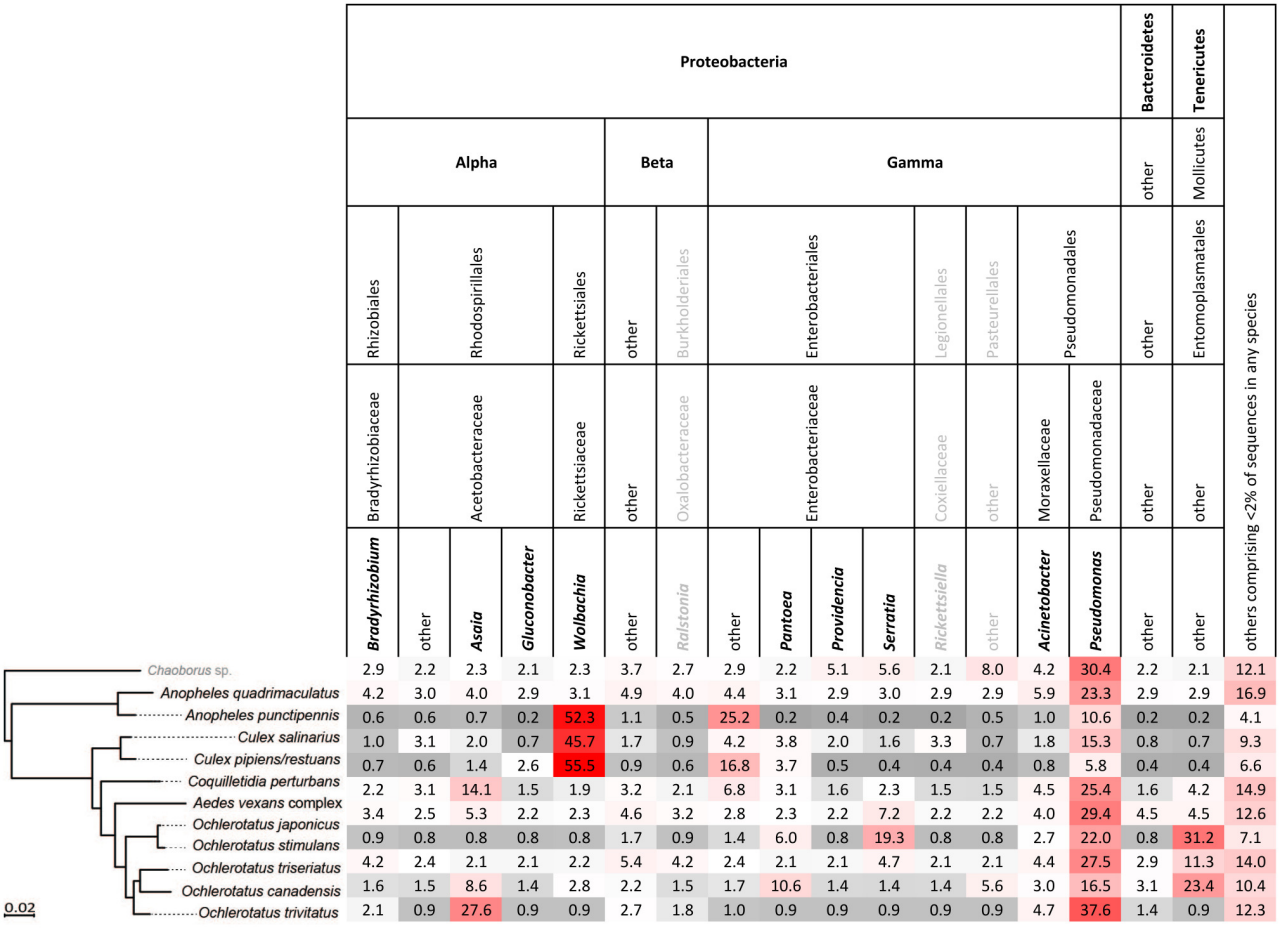
**Relative abundance of bacterial taxa in each host species arranged in order of phylogenetic relationship, based on single mosquito samples**. Bold printed taxa were congruently found in abundances above 2% for the pooled samples (Figure S2).

**Figure 4:**
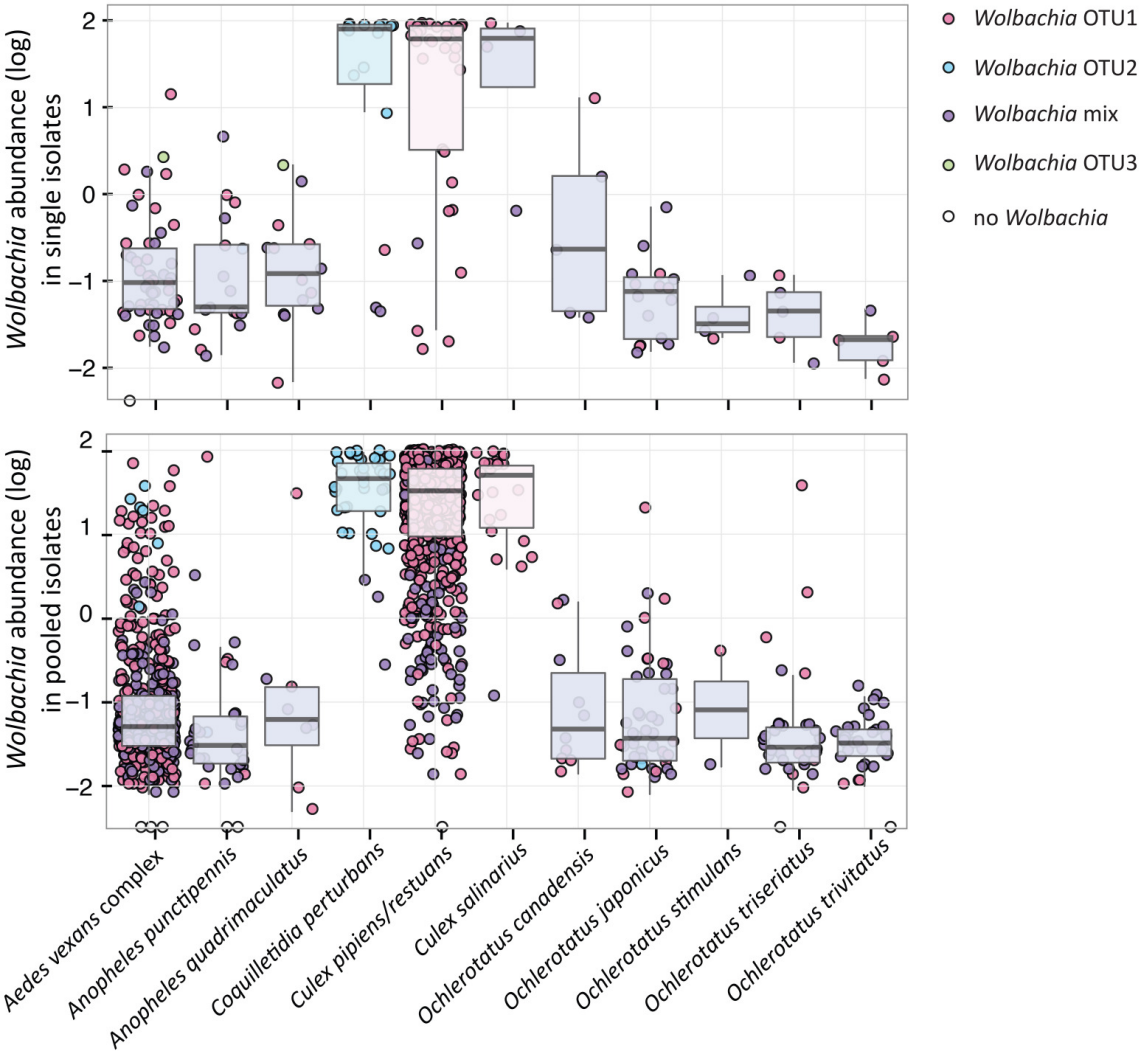
*****Wolbachia*** relative abundance and presence of dominating OTUs for 11 analyzed species sampled as individuals and pools**. The relative abundance represents 16S rRNA read percentage assigned to any *Wolbachia* OTU. The color key for individual points (samples) and box plots reflects the presence of particular OTU(s), i.e., 100% of reads assigned to the single *Wolbachia* OTU in red, blue and green; mixed infection in purple.

**Figure 5:**
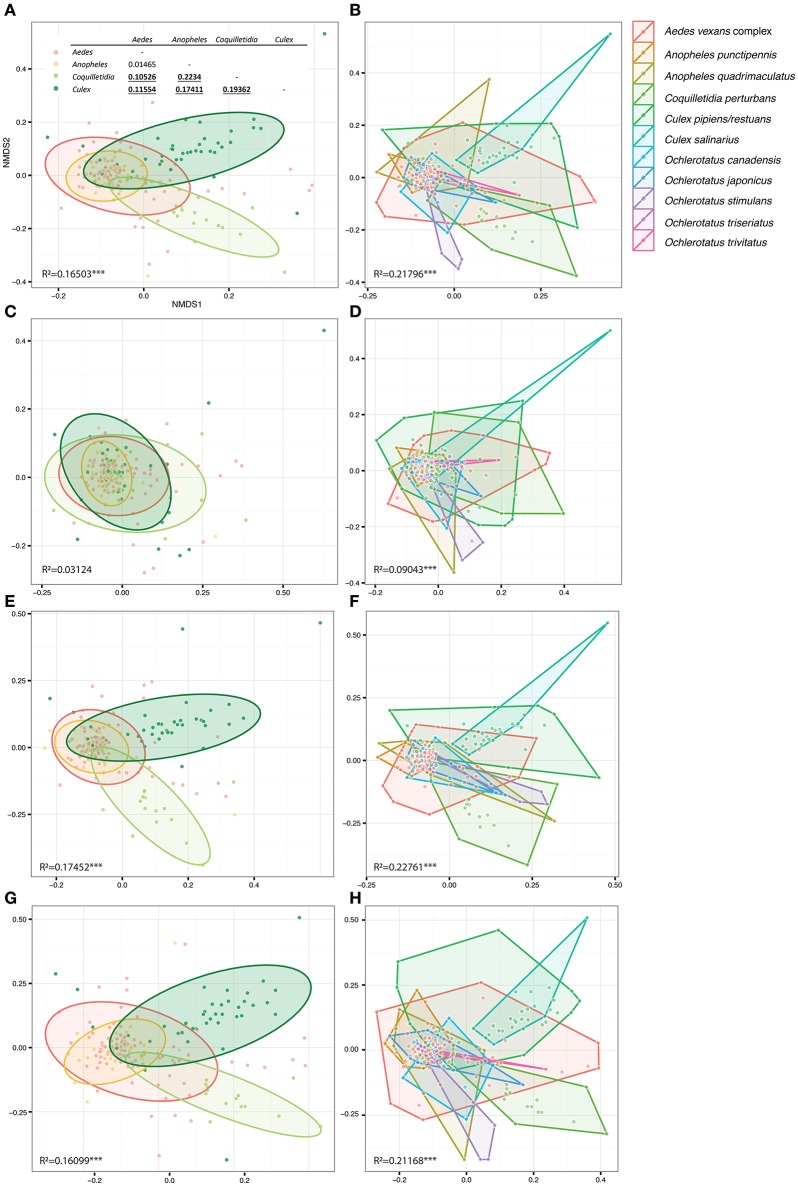
**Overall differences among microbiomes of different mosquito genera (right) and species (left) plotted in constrained ordinations**. **(A,B)** were produced using the complete single mosquito dataset; **(C,D)** are based on data from which all *Wolbachia* OTUs were removed in order to test *Wolbachia* effects on dissimilarity among microbiota profiles (for more details see Materials and Methods). **(E,F)** present control analyses excluding the next most abundant OTU, i.e., *Asaia* **(E,F)**, from the data set, and an OTU shared by all the mosquito taxa, i.e., *Pseudomonas* **(G,H). (A)** includes pairwise statistical evaluation: bold underlined numbers stand for *R*^2^-values significant at 99% confidence interval calculated for dissimilarities of genera pairs. *R*^2−^values indicate statistical evaluation of dissimilarities among all genera/species in each plot. Considering the low number of samples per species, hulls were used to highlight the corresponding points, instead of the statistical ellipses used for genera based analyses.

Single-mosquito samples were different than pooled samples (noted hereafter as ^**^ for 95% confidence and ^***^ for 99% confidence) in diversity of mosquito microbiota (Mann-Whitney *U*-test for richness: *U* = 107,639^***^; Mann-Whitney *U*-test for Shannon diversity index: *U* = 144,615^**^). The average total read number for individuals was 42,990, and 82,124 for pooled samples. The mean (SD) bacterial richness in microbiota of all 11 species was 50.9 (17.3) bacterial OTUs for single-mosquito samples and 64.8 (31.3) bacterial OTUs for pooled samples (Figure 2). Highly abundant taxa found to be associated with at least one of the analyzed mosquito species were primarily of the phylum Proteobacteria, including *Asaia, Wolbachia, Serratia, Pseudomonas* and other bacteria from the family Enterobacteriaceae. Except for the Proteobacteria, members of Entomoplasmatales (Tenericutes) were also found in high numbers in some *Ochlerotatus* species. Relative abundances of these principal bacterial taxa calculated for single-mosquito samples are shown in Figure 3 (Figure S2 pooled samples). There were 36 OTUs that differed significantly among species based on Kruskal-Wallis test with FDR correction (Supplemental data: group_significance_results.xlxs). There were significant differences among the microbiota of different host species using Bray-Curtis dissimilarity matrices, and differences were less distinct after removal of *Wolbachia* symbionts (Figures 4, 5).

### Phylosymbiosis analysis

**Figure 6:**
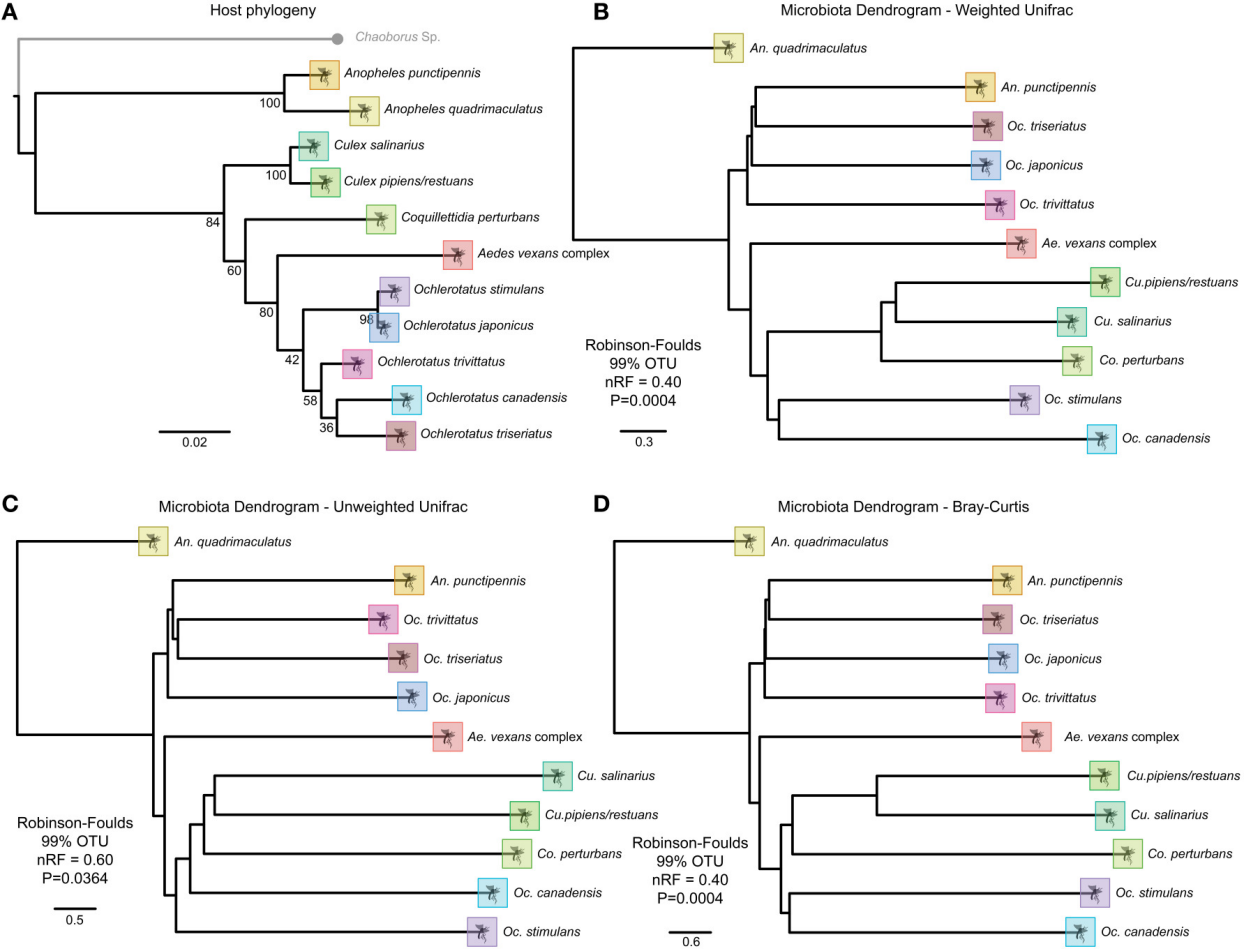
**Topographic trees of host phylogeny and OTU beta-diversity metrics indicating strong phylosymbiosis of mosquito host species and microbiota. (A)** RaxML host phylogeny based on an incomplete multigene matrix of 18S, 28S, COI and NADH. Beta-diversity analysis for each host species at 99% OTU clustering for **(B)** Weighted UniFrac, **(C)** Unweighted UniFrac, and **(D)** Bray Curtis metrics.

All beta-diversity distance matrices indicated an accurate separation of the *Anopheles* genus, and some conservation of phylosymbiosis between major genera is maintained when average bacterial communities are clustered at 97 or 99% OTU identity (Figure 6). The relationship of the host phylogeny and the 97% OTU clustering of microbial communities is nearly completely incongruent with the exception of the weighted unifrac (RF index of 0.75, Table S3). However, as recently observed in Brooks et al. (2016), when microbial communities are clustered at 99% OTU identity, all beta-diversity analyses conducted indicate significant phylosymbiosis for the wild mosquito species and their respective microbial communities (Table S3).

### Wolbachia

Within the 11 species analyzed, three (*Cx. pipiens/restuans, Cx. salinarius* and *Cq. perturbans*) were found to harbor *Wolbachia* in high numbers (Figure 4). In *Cx. pipiens* pooled samples (*n* = 591), *Wolbachia* was not detected in one sample (a 6 individuals pool sampled in June 2011), indicating a high prevalence in this mosquito species. A total of 17 *Wolbachia* OTUs (reduced to 13 by phylogenetic analysis, Figure S3) were found among the 11 mosquito species, but only 3 were found at high abundance. Ten species were associated with *Wolbachia* OTU1 or a mixed infection, *Coquilletidia perturbans* harbored a distinct *Wolbachia* strain (represented by *Wolbachia* OTU2 with 94.2% similarity in 125 bp to OTU1, Figure 4, Figure S3). *Wolbachia* symbionts dominated microbial communities of *Culex* and *Coquilletidia* species profiled in Figures 5A,C. Comparing microbiota at the host genus level with pairwise comparisons revealed significant differences between all the pairs, except for the *Aedes*-*Anopheles* pair (Figure 5A; bold underlined numbers stand for Adonis *R*^2^-values significant at the 99% level). In contrast, using the filtered dataset lacking all *Wolbachia* OTUs, no significant differences were found among the mosquito genera pairs (Figure 5). Statistical evaluation of differences between mosquito species pairs for complete and *Wolbachia* free datasets is provided in Table S4, highlighting the distinctive effect posed particularly on the microbiome profiles of *Cx. pipiens/restuans* and *Coquilletidia perturbans* by these bacteria.

### Biogeography and seasonal effects on mosquito microbiota

The effect of geographical background on the microbiota was tested for pooled samples of *Ae. vexans* complex and *Cx. pipiens/restuans*, the two taxa with sufficient sample sizes for statistical evaluation. Because we did not find significant differences among years, tests for biogeographical effect were performed over the 3-year period. Site of capture did not significantly affect microbiota of *Ae. vexans* complex pooled samples from six different regions (Figure 1; Adonis: *R*^2^ = 0.00845). Similarly, site was not a significant factor differentiating microbiota from single *Ae. vexans* complex samples from Brant and Toronto (*R*^2^ = 0.0659). Pooled samples from *Cx. pipiens/restuans* allowed for testing among nine sites. The analyses produced significant results for differences between following regions: Brant-Windsor Essex (*R*^2^ = 0.12065^***^), Peterborough- Windsor Essex (*R*^2^ = 0.2948^***^) and Haldimand- Windsor Essex (*R*^2^ = 0.17541^***^). Windsor Essex was the most distinct site and at the edge of the sampling region (Figure 1).

**Figure 7:**
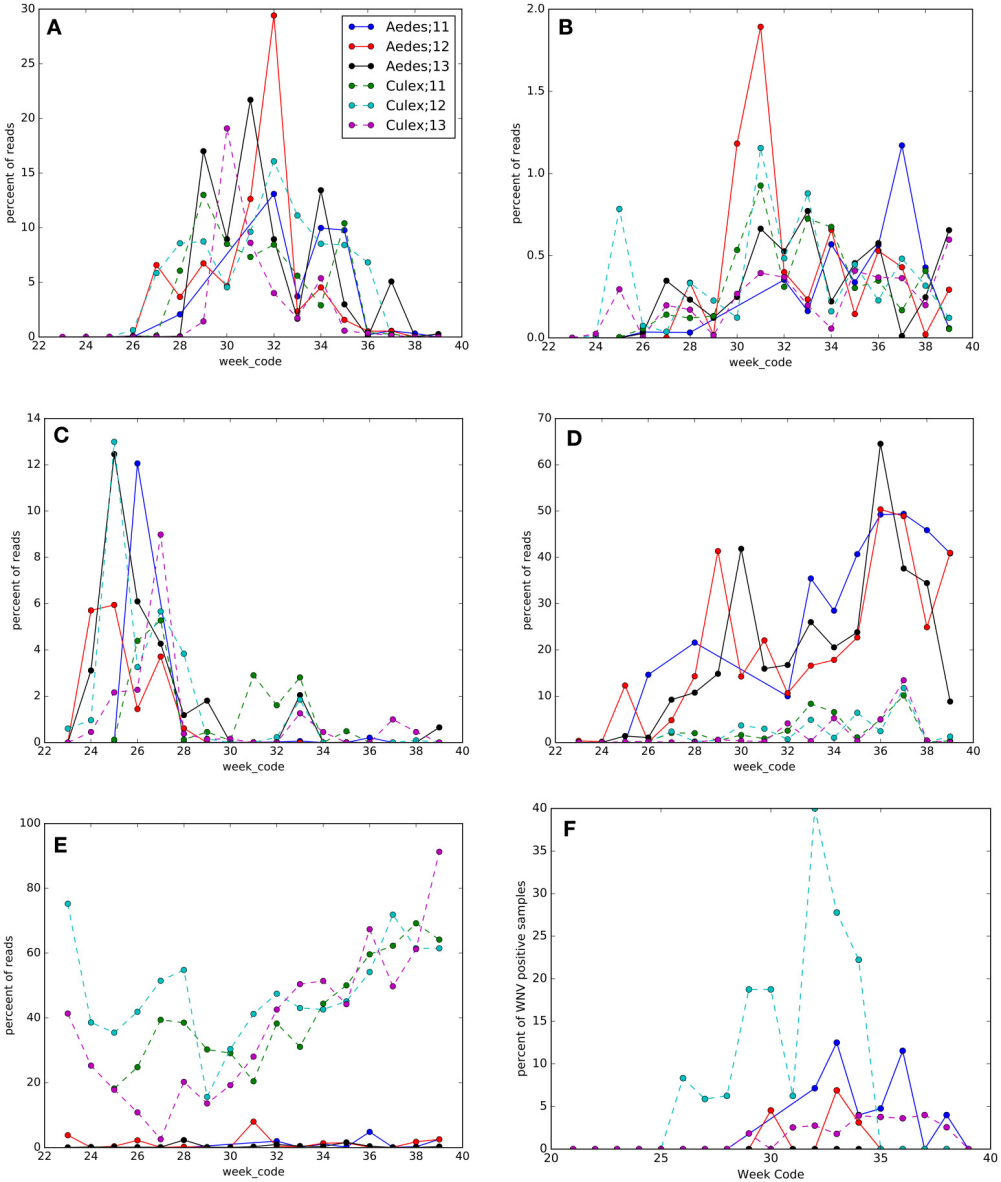
**Seasonal changes in OTUs and West Nile virus (WNV) prevalence in ***Culex pipiens/restuans*** (Culex) and ***Aedes vexans*** complex (Aedes) pools sampled between 2011 and 2013 (see key for species and year)**. Taxonomy of OTUs in each panel: **(A)** Proteobacteria; Alphaproteobacteria; Rhodospirillales; Acetobacteraceae, **(B)** Bacteroidetes, **(C)** Proteobacteria; Gammaproteobacteria; Enterobacteriales; Enterobacteriaceae, **(D)** Proteobacteria; Alphaproteobacteria; Rhodospirillales; Acetobacteraceae; *Asaia*, **(E)** Proteobacteria; Alphaproteobacteria; Rickettsiales; Rickettsiaceae; *Wolbachia*, and **(F)** WNV prevalence.

Along with seasonality in mosquito density (Table S1, Figure S1), we found overall seasonal fluctuations in the microbiota in *Ae. vexans* complex and *Cx. pipiens/restuans* (Figure 7, Table S5, Figures S4, S5). The OTUs with the greatest seasonal dynamics (largest effect sizes) are indicated in Table S5, with trends illustrated in heatmaps for each species (Figures S6, S7). The abundance of *Wolbachia* shifted seasonally in *Cx. pipiens/restuans* with a dip in June-July, but not in *Ae. vexans* complex (Figure 6E). Four other OTUs showed seasonal trends in each of the 3 years sampled both in *Cx. pipiens/restuans* and in *Ae. vexans* complex including Acetobacteraceae, Bacteroidetes, Enterobacteriaceae, and *Asaia* (Figure 6). Seasonal dynamics were not analyzed for the 9 other host species with smaller sample sizes.

### Seasonal changes in WNV prevalence and microbiota

Sampling mosquitoes for WNV and microbiota across a 3 year period in Ontario revealed 6 species as potential vectors for WNV (Table 1), with the highest prevalence in the *Cx. pipiens/restuans* and a seasonal spike in prevalence each year in early to mid August reaching up to 43% of pooled samples (Figure 7F). Species exhibiting low relative abundance of *Wolbachia*, including all *Ochlerotatus, Aedes* and *Anopheles* specimens, were identified as potential WNV carriers. Samples of *C. perturbans* associated with *Wolbachia* OTU2 in high densities and were found in other studies to have low WNV infection prevalence (Sardelis et al., 2001; Cupp et al., 2007). Out of seven species with single mosquito samples showing some WNV positives, 6 species had higher mean abundance of *Asaia*, and 7 species had higher mean abundance of *Wolbachia* in WNV uninfected compared to infected mosquitoes. In *Cx. pipiens/restuans* samples, mean *Wolbachia* reads were approximately 68.8% (*N* = 31) in WNV negative samples compared to 0.3% (*N* = 3) in WNV positive samples. There was a dip in *Wolbachia* prevalence and a nearly corresponding spike in WNV prevalence in pooled samples of *Cx. pipiens/restuans* (Figure 7). Conditions in the weeks prior to sampling were critical in driving patterns of WNV.

**Figure 8:**
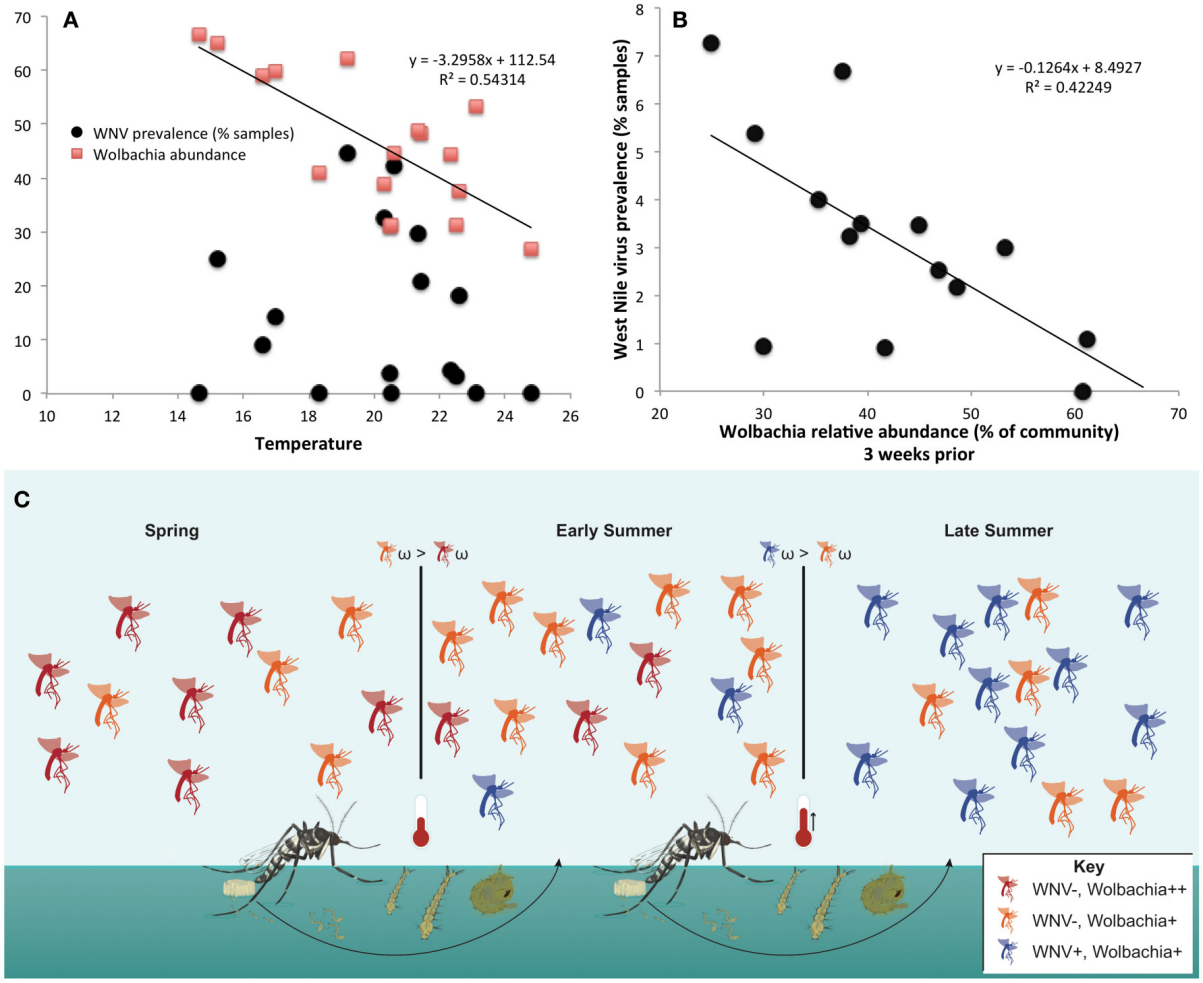
**Temperature negatively correlates with ***Wolbachia*** abundance, and ***Wolbachia*** abundance negatively correlates with West Nile virus (WNV) in ***Culex pipiens/restuans*****. **(A)** *Wolbachia*, but not WNV, is significantly correlated with mean weekly temperature in samples (*n* = 538) from Toronto. Daily climate data for 2011–2013 Toronto (WMO identifier 71265) downloaded from: http://climate.weather.gc.ca/historical_data/search_historic_data_e.html. **(B)** The relative abundance of Wolbachia in Cx. pipiens/restuans pooled samples (*n* = 591) over 3 years was significantly correlated with West Nile virus prevalence in pooled samples after a 3 week delay (see Table S6). Note that although the correlation coefficient increased from weeks 0 to 3, there was not a significant correlation until 3 weeks had elapsed. Thus, a decrease in Wolbachia abundance may contribute to conditions favoring WNV susceptibility in the succeeding mosquito generation. **(C)** A conceptual model illustrates the hypothesis that higher temperatures reduce Wolbachia abundance (orange) and lead to higher WNV prevalence (blue) in the following generation. Subsequently, fitness costs of WNV (ω), seasonal reductions in temperature, or lower density of mosquito larvae may drive the cyclical pattern and selection for increased abundance of protective Wolbachia (red).

*Wolbachia* abundance in *Cx. pipiens/restuans* pooled samples negatively correlated with temperature (Figure 8A). There was a significant correlation between *Wolbachia* abundance 3 weeks before sampling and WNV prevalence (*R*^2^ = 0.42249, *P* = 0.012, Figure 8B). The correlation coefficient increased with time prior to sampling for WNV prevalence vs. temperature and WNV vs. *Wolbachia* abundance; precipitation did not correlate with WNV prevalence (Table S6). Temperature negatively correlated with *Wolbachia* abundance (Figure 8A), and temperature 3–4 weeks prior to sampling correlated with WNV prevalence (Table S6). Thus, higher temperatures may have led to decreased *Wolbachia*; through vertical transmission to the subsequent generation, reduced *Wolbachia* is hypothesized to increase susceptibility to WNV (Figure 8C). Given that a 2°C increase in peak summer temperature would decrease *Wolbachia* abundance by 22% (Figure 8A), this reduction of *Wolbachia* could lead to an 18% increase in WNV prevalence from 4.7 to 5.5% of samples positive (Figure 8B). This scenario of climate change is realistic in eastern North America, particularly in urban areas (Primack, 2014). We suggest that WNV prevalence in *C. pipiens/restuans* may increase in samples collected after sampling for this study completed in 2013.

## Discussion

Recent microbiome studies focus on factors driving the composition and function of host microbiota. In this study, we examined 11 adult mosquito host species from six regions in southern Ontario, Canada. Mosquitoes were sampled over 3 years in the Toronto region. We found that host species was the largest driver of the microbiota, while region had little impact for the species tested (*Cx. pipiens/restuans* and *Ae. vexans*). However, the region with the most distinct microbiota, Windsor Essex, was at the edge of the sampling region, indicating that over larger geographical scales than studied here, region may be an important factor driving microbiomes, or that region is correlated with important environmental conditions. Seasonal shifts were consistently repeated over the 3-year period in microbiomes of *Cx. pipiens*/*restuans* and *Ae. vexans* complex. Both host species and seasonal shifts in microbiota correlate with patterns of WNV in these mosquitoes.

In accordance with previously published results on *Anopheles* and *Culex* genera (e.g., Gimonneau et al., 2014; Duguma et al., 2015), we found that microbiota of *Aedes, Ochlerotatus, Anopheles, Culex*, and *Coquilletidia* species were dominated by the phylum Proteobacteria. This common pattern suggests that some characteristics of the Proteobacteria may make them especially suitable for mosquito colonization. Interestingly, although a clear environmental influence from the water stages to the adults has been detected (Coon et al., 2014; Tchioffo et al., 2016), the microbiota seems to differ specifically between mosquito genera or even between species within the same genus (e.g., Muturi et al., 2016) regardless their origin, suggesting a certain level of selection toward a beneficial microbiota (Gimonneau et al., 2014). Indeed, several predominant Proteobacteria have been found to have protective affects on mosquitoes including *Serratia* (Bando et al., 2013; Tchioffo et al., 2016) and *Wolbachia* (Moreira et al., 2009). Abundant *Pseudomonas* are commonly found across mosquito species (Charan et al., 2013; Minard et al., 2013). The presence of at least some bacterial strains, regardless of their origin, may be essential for successful mosquito development (Chouaia et al., 2012; Coon et al., 2014), digestion, and fecundity (Gaio et al., 2011).

This is the first observation of wild caught mosquitoes exhibiting phylosymbiosis under natural conditions. In the recent study by Brooks et al. (2016), laboratory reared mosquito species were isolated in near identical conditions without access to natural microbial communities or *Wolbachia* infections. In the study presented here, we observed significant (*p* < 0.005) similarities between the host species phylogeny and the microbial community composition within a given species using the same analysis. This strengthens the hypothesis that there is host selection on the microbial communities between species that is independent of environmental factors.

The present study brings further insight into microbiota of less-studied mosquito genera, i.e., *Ochlerotatus* and *Coquilletidia*, and reveals significant differences among all analyzed species and genera. These differences were mainly driven by the presence and abundance of *Wolbachia*, the widespread intracellular symbiont with an immense diversity of strains and phenotypes (e.g., Werren et al., 2008). Indeed, two different strains of *Wolbachia* were found in high numbers in *Culex* and *Coquilletidia* species. *Wolbachia* dominance is particularly highlighted when removing individual *Wolbachia* OTUs from the analyses. Differences among and between most species/genera become insignificant when *Wolbachia* is excluded. While *Cx. pipiens/restuans* and *Wolbachia* wPip experienced a common evolutionary history (e.g., Atyame et al., 2011), *Wolbachia* symbionts may represent the selective force shaping the rest of the microbial community resulting in the exclusive characteristics of the entire system.

One recent study (Muturi et al., 2016) provided comparative results on microbiota of *Culex* species showing significant differences in relative abundance of dominant bacteria in *Cx. pipiens* and *Cx. restuans*. While our sample collection and analysis clustered together specimens in this morphologically indistinguishable species complex, their microbiota are more similar to those of *Cx. restuans* (56% Alphaproteobacteria and 21% Gammaproteobacteria), compared to *Cx. pipiens* (94% and 4% relative abundance, respectively) found by Muturi et al. (2016). In addition, Muturi et al. (2016) showed over 90% relative abundance of *Wolbachia* in *Cx. pipiens* from central Illinois compared to 47% in the species complex described here from southern Ontario.

Our results indicated four main bacterial genera dominating the analyzed microbiota, namely *Wolbachia, Asaia, Serratia* and *Pseudomonas*. Isolates of *Serratia* have been associated with anti-*Plasmodium* effects in some *Anopheles* mosquitoes (Bando et al., 2013; Tchioffo et al., 2016). *Wolbachia* and *Asaia* symbionts have also been previously described from other adult mosquitoes (*Asaia* in different *Anopheles* species, Crotti et al., 2009; *Wolbachia* in several *Aedes* and *Culex* species, Sunish et al., 2011; Lu et al., 2012; Bian et al., 2013; Sinkins, 2013; Dutra et al., 2016; Muturi et al., 2016). These later two genera were demonstrated to be mutually exclusive, and in some mosquito species *Asaia* can prevent *Wolbachia* infection and vice versa (Rossi et al., 2015). Our results in suggest a similar trend of mutual exclusion in *Ae. vexans* complex and *O. trivitatus*. Additionally, the high abundance of *Pseudomonas* in *Anopheles, Ochlerotatus* and *Aedes* may also exclude the presence of *Wolbachia* in the system, suggesting that bacteria other than *Asaia* may affect the ability of the system to retain stable *Wolbachia* infection (Hughes et al., 2014). This is particularly relevant for disease transmission by mosquito vectors, as *Wolbachia* has been linked to vector competence (e.g., Micieli and Glaser, 2014).

In fact, a range of effects posed by *Wolbachia* on pathogens and parasites has been described for different insect hosts (Lu et al., 2012; Dodson et al., 2014). Particularly, some *Wolbachia* strains in combination with certain hosts are protective against viruses, but not others, as it happens in *Drosophila* (Osborne et al., 2009; Faria et al., 2016). In those cases of specific protective combinations, *Wolbachia* are found in higher densities compared to systems with non-protective *Wolbachia* phenotypes (i.e., in mosquitoes: Lu et al., 2012; Bian et al., 2013; and fruit flies: Osborne et al., 2009; Faria et al., 2016). These findings led some authors to the hypothesis that all *Wolbachia* strains are capable of antiviral protection if a sufficient density is reached, although that density level may be dependent on the host compatibility (e.g., Johnson, 2015). Here, with the presented data for *Cq. perturbans* and *Cx. pipiens/resturans* we question this general hypothesis. Both species, being capable of the WNV transmission (Sardelis et al., 2001), harbor different strains of *Wolbachia* in comparable abundances (median value calculated for single isolates are 78% for *Cq. perturbans* and 62% for *Cx. pipiens/restuans*, Figure 4). While 8.1% of 3,648 pooled *Cx. pipiens/restuans* samples were found positive for WNV, *Cq. perturbans* is not a priority species for WNV surveillance based on low field prevalence and low vector competence (Sardelis et al., 2001; Cupp et al., 2007). This further highlights the importance of host genetic background and *Wolbachia* strain combination, along with the symbiont abundance, as the main factors underlying host WNV carrier status, and vector competence. Our data, being generated from entire mosquito bodies, provide relative approximations on total *Wolbachia* numbers. The outcome of viral exposure may, however, depend on particular cellular or tissue levels of *Wolbachia* at the virus replication sites. Future research endeavors could thus combine the high-throughput population surveys with fluorescent *in situ* hybridization (FISH) approaches in order to localize and precisely quantify *Wolbachia* cells.

The microbiota of particular mosquito species may be an outcome of several factors. These include (i) host genetic background, (ii) long-term interactions among the bacteria and/or (iii) mutual interplay between host, microbiota and transmitted pathogen. This can be illustrated by recent findings of Martinez et al. (2015) on *Wolbachia* strain variation in terms of beneficial antiviral protection and parasitic cytoplasmatic incompability (CI). Strains that conferred antiviral effects negatively affected life-history traits and had a fitness cost compared to strains with CI in *Drosophila simulans*. Thus, persistence of antiviral *Wolbachia* strains in a mosquito population may depend on the prevalence and the burden of viral infections. Although few studies have examined burdens to mosquitoes of their vectored viruses, WNV caused increased mortality of *Cx. pipiens* and had strain-specific effects on fecundity and blood feeding behavior (Ciota et al., 2013).

In our data, we found a striking difference of WNV prevalence in two major vectors. Compared to an estimated 8.1% of WNV positive *Cx. pipiens/restuans* sample pools, the estimated WNV prevalence in populations of *Ae. vexans* complex is much lower (<1%). Low abundance of *Wolbachia* in this species indicates that other microbiota members, particularly *Asaia* and *Pseudomonas*, may confer antiviral protection with less fitness costs. Alternatively, apparently lower susceptibility to WNV infection may stem from host genetics, or differences in host feeding ecology.

Vector competence trials repeatedly demonstrate differences among strains of virus, and also among species and populations of vector mosquitoes. For example, *Ae. aegypti* from Santiago Island, Cape Verde exhibited high vector competence for DENV-2 and DENV-3 serotypes and a low susceptibility to DENV-1 and DENV-4 (da Moura et al., 2015). Variable population susceptibility to dengue virus has been attributed to differences in immune transcription (Carvalho-Leandro et al., 2012). Vector competence for the Asian genotype of Zika virus differed between populations of *Ae. aegygti* and between species *Ae. aegypti* and *Ae. albopictus* (Chouin-Carneiro et al., 2016). In addition to genetic differences among populations, we hypothesize that differences in vector competence are also caused by differences in microbiota affecting immune gene expression.

*Wolbachia* is one symbiont, among others, with known immuno-modulatory capacity in mosquitoes linked to vector competence (Kambris et al., 2009; Jupatanakul et al., 2014; Hegde et al., 2015). Here, we examined environmental effects on the microbiome as a potential mechanism for viral pathogen regulation, and found a striking correlation of season and temperature in particular that may regulate *Wolbachia* abundance in *Cx. pipiens/restuan*s hosts. *Wolbachia* abundance, in turn, may impact susceptibility to WNV infection status and prevalence of WNV at later time points. In experimental studies, as temperature was increased from 14 to 30°C, there was an increase in WNV titer in *Cx. tarsalis* (Reisen et al., 2006), indicating that climate can play an important role in disease dynamics. Temperature increases are known to reduce *Wolbachia* abundance across mosquito life stages (Wiwatanaratanabutr and Kittayapong, 2009; Ye et al., 2016). Ciota et al. (2014) examined life history traits of *Culex* mosquitoes. They found that days to emergence could range from approximately 25 to 12 days depending on temperatures of 16–24°C, respectively. Larvae with reduced *Wolbachia* could be sampled as adults as early as 2–3 weeks later depending on temperature, or adults with reduced *Wolbachia* could reproduce, and transmit a low abundance of *Wolbachia* to the next generation in that timeframe. Thus, reductions in protective microbiota mediated by climate warming in addition to increased viral replication (Dohm et al., 2002) may lead to increased WNV and other arboviruses in both vertebrates and their mosquito disease vectors. Alternatively, independent of the seasonal changes in *Wolbachia* that are correlated with mean temperature, there may be an increase in WNV prevalence caused by a seasonal increase in infected blood-meal hosts.

A general role of seasonality has previously been suggested to affect microbial abundance in other blood sucking vectors including fleas and ticks (Lalzar et al., 2012; Cohen et al., 2015). In fleas, the spring-to-summer changes found in the bacterial community were attributed to the compositional changes in the diet, i.e., blood, including presence of pathogens (Cohen et al., 2015). The present study lacks the information on blood meal origin. However, considering seasonal fluctuations in bird populations, the preferred mosquito host and the reservoir for WNV, variation in the blood meal seems a plausible explanation for WNV seasonality. Additional environmental conditions may be responsible for seasonal effects detected in other microbiota. Some OTUs peak mid-summer such as Acetobacteraceae or Bacteriodetes, others such as Enterobacteriacea decrease yearly, or like *Asaia*, increase yearly, while other OTUs have fluctuating trends.

We found a strong seasonal pattern in WNV prevalence repeated over 3 years in *Cx. pipiens/restuans* mosquitoes. We also found seasonal patterns in other microbiota, indicating a potentially broad role for microbiota in pathogen defense and vector competence. This likely extends beyond *Wolbachia*, the dominant seasonal member in *C. pipines/restuans*, and current focus for disease mitigation against Flaviviruses (Dutra et al., 2016). Indeed, extended immunity provided directly by microbiota may be a trait under selection (Correa and Ballard, 2016; Faria et al., 2016), particularly if harboring pathogens has a fitness cost to the mosquitoes, as it does for WNV (Ciota et al., 2013). With increasing prevalence of mosquito-borne viruses there will be increased selection pressure on mosquitoes for symbiotic microbiota that increase resistance to viruses.

The most successful use of microbial management of insect vectors has been the application of *Bacillus thuringiensis* serotype *israelensis* (*Bti*) as a larvicide to reduce black fly populations in Western Africa to control onchocerciasis (Mbewe et al., 2014). *Bti* is now the only insecticide permitted in many European countries for mosquito control (Paris et al., 2011a) and *Bti* has become increasingly employed in mosquito control programs in the USA (Floore, 2006). Although resistance to other strains of *Bacillus thuringiensis* has been shown for several insect groups, the appearance of resistance to *Bti* toxins in natural vector populations has only recently been found in mosquitoes under some circumstances (Paris et al., 2011b; Bonin et al., 2015; Stalinski et al., 2014) but not in others (Araújo et al., 2013). The potential for such evolution presents a concern, and may be inevitable if *Bti* use becomes more prevalent. Like antibiotic resistance, a consistent use of chemical insecticides, including *Bti* toxins, sets the stage for selection in favor of resistant genotypes. We found that some *Bacillus* taxa increase seasonally (Table S5), and hypothesize that this may be influenced by applications of *Bacillus* larvicides and evolving resistance among *Cx. pipiens*. Indeed, an OTU matching a commonly used larvicidal agent, *Lysinibacillus sphaericus* (e.g., Valent Bioscience's VectoLex http://publichealth.valentbiosciences.com/products/vectolex), was found here on adult mosquitoes, although detection of genes involved in toxicity (i.e., Guidi et al., 2013) are needed to determine whether mosquitoes may be developing resistance to the larvicide.

## Conclusion

The species analyzed here harbor significantly different microbial communities, all dominated by Proteobacteria. In this 3-year field survey we examined factors influencing the dynamics of mosquito microbiota. We found that host genetic background explained most of the variation, followed by season and geographic region as important drivers of the microbiome, similar to findings from other animal groups (e.g., Kueneman et al., 2014). Coevolution, and thus functional importance of the microbiome, is indicated by the relatedness of microbial communities of mosquito hosts in parallel to the host phylogeny (phylosymbiosis). A long-term coevolutionary relationship between *Wolbachia* and some host species may strongly influence the structure of the rest of the bacterial community. The presence of *Asaia* and *Pseudomonas* fluctuates with the presence of *Wolbachia* in mosquito hosts, supporting this hypothesis. The dynamic background of mosquito microbiota described here may help explain epidemiological patterns of WNV. For instance, if increasing temperatures cause a decrease in protective *Wolbachia*, climate warming may escalate disease caused by WNV. The importance of microbiota mediated by global change may have ramifications for mosquito-borne pathogens that are just beginning to be explored.

## Author contributions

EN and DW contributed equally to this manuscript. DW, RK, JS, and EN contributed to the original idea; JS, AM, and DW contributed to field collection or lab analyses; DW, EN, SR, AA, RB, JL, JS, and AM contributed to data analyses. DW, EN, SR, RB, and RK wrote and revised the manuscript.

## Funding

This project was partially funded by the Earth Microbiome Project, and the UMass Boston Proposal Development Grant Program to DW.

### Conflict of interest statement

The authors declare that the research was conducted in the absence of any commercial or financial relationships that could be construed as a potential conflict of interest.
