# Supplementary material for: Mosquito Microbiome Dynamics, a Background for Prevalence and Seasonality of West Nile Virus
Source: Front Microbiol. 2017 Apr 4;8:526. doi: 10.3389/fmicb.2017.00526 (PMC5378795; doi:10.3389/fmicb.2017.00526)
Supplement: Supplementary file 2 [file DataSheet2.docx]

Supplemental material for

**Mosquito Microbiome Dynamics, a Background for Prevalence and Seasonality of West Nile Virus**

Eva Novakova, Douglas C. Woodhams, Sonia M. Rodríguez-Ruano, Robert M. Brucker, Jonathan W. Leff, Amin Maharaj, Amnon Amir, Rob Knight, James Scott

**Contents:**

**Supplemental Methods**

**Supplemental Tables**

**Table S1.** Mosquito samples analyzed from 2011-2013 from Ontario, Canada.

**Table S2.** Primer and probe sequences for real time TaqMan PCR for West Nile virus detection from mosquitoes.

**Table S3**. Topological congruencies of phylosymbiosis between host phylogeny and microbiota dendrogram

**Table S4.** Statistical evaluation of pairwise differences between microbial communities of mosquito hosts.

**Table S5**. OTUs with significant seasonal trends in *Aedes vexans* comples and *Culex pipiens/restuans*.

**Table S6**. West Nile virus tested for correlation with mean air temperature, precipitation, and *Wolbachia* abundance in ambient conditions or conditions from prior sampling weeks.

**Supplemental Figures**

**Figure S1.** Number of mosquitoes captured each week.

**Figure S2**. Heatmap describing relative abundance of bacterial taxa in pooled samples of 11 mosquito species.

**Figure S3.** Maximum likelihood based phylogenetic relationships among all *Wolbachia* OTUs found in the complete dataset.

**Figure S4.** Beta-diversity plots showing seasonality of microbiota in *Aedes vexans*

**Figure S5.** Beta-diversity plots showing seasonality of microbiota in *Culex pipiens*.

**Figure S6.** Heatplots of OTUs showing wavelike seasonal patterns of abundance.

**Figure S7.** Heatplots of OTUs showing wavelike seasonal patterns of abundance.

**Supplemental References**

**Supplementary Methods**

*Mosquito sampling*

Mosquito samples analyzed here are shown in Table S1. Only data from Toronto is available for each year (2011-2013) from June to September. All data from 2011 is from Toronto sampled on a weekly basis for 15 consecutive weeks (epidemiological weeks 25-39) from June to September. In 2012, Toronto was sampled on a weekly basis for 17 consecutive weeks (epidemiological weeks 23-39). Additionally in 2012, the regions of Brant, Lambton, Middlesex-London, Peel and Windsor-Essex were sampled for 5 consecutive weeks (epidemiological weeks 30-34) from July to August. In 2013, Toronto was sampled on a weekly basis for 17 consecutive weeks (epidemiological weeks 23-39) from June to September. Additional regions sampled in 2013 include; Haldimand-Norfolk (2 weeks, epidemiological weeks 31 and 33, July and August); Lambton (4 consecutive weeks, epidemiological weeks 35-38, August to September); Niagara (2 weeks, epidemiological weeks 29 and 34, July and August); and Peterborough (6 weeks, epidemiological weeks 31-35 and 37, August to September).

Traps (CDC ultraviolet light traps) were operational for a 24 hour period, then transported overnight to the laboratory in coolers with the exception of traps collected in Toronto which were delivered on the day of collection. After arrival at the laboratory, traps containing live mosquitoes were incubated at -80°C for 20 minutes. Specimens were then removed and placed on ice blocks for morphological identification using stereo microscopes with fiber optic and halogen illumination and cold stage ability. Up to 150 female mosquitoes were arbitrarily grab-sampled from each trap. Non-mosquito species and male mosquitoes were discarded. Mosquitoes were identified by species using a dichotomous key (Darsie and Ward, 2005, Thielman and Hunter, 2007). Pools of the three highest priority species were tested per trap. Species priority was mandated by the Ontario Ministry of Health and is based on vector competence. Up to 50 mosquitoes of the same species were added to a pool in 2mL conical polypropylene screw cap tubes containing one sterile 4.5mm diameter steel ball bearing (BB-pellet).

Mosquito pools were homogenized in 1mL of BA-1 diluent (1 x medium 199 with Hanks’ balanced salt solution, 0.05M Tris pH 7.5, 1% bovine serum albumin, 3.5g/L sodium bicarbonate, 100µg/mL streptomycin, 1µg/mL amphotericin B) modified from the protocols of Lanciotti et al. (2000). Samples were homogenized in the FastPrep®-24 (MP Biomedicals) at a setting of 6.0 for 60 seconds. Following centrifugation, 200 µL of supernatant was used for RNA extraction. RNA extraction was performed using either the E-Z 96® Total RNA Kit II (96 well-plates; Omega Bio-tek Inc., Norcross, GA) or the E.Z.N.A® Total RNA Kit I (individual tubes) from Omega Bio-tek following the manufacturer’s instructions. Sample homogenate was stored long-term at -80°C.

*West Nile virus detection*

A total of 5µL of RNA extract was used in a 20µL reaction with the iTaq^TM^ Universal Probes One-Step kit, containing 1µM of each primer and 0.2µM of probe (Bio-Rad Laboratories, Hercules, CA). The thermal cycling consisted of 50°C for 10 minutes, 95°C for 5 minutes, and 40 cycles of 95°C for 15 seconds and 60°C for 1 minute, performed in a Chromo4 real time PCR detector (DNA Engine, Bio-Rad) or a CFX Connect Real-Time PCR Detection System (Bio-Rad). If a positive pool was detected, RNA was processed in a second real time PCR assay for confirmation using the WNENV primers and probes (see text).

**Supplemental Tables**

**Table S1.** Mosquito samples analyzed from 2011-2013 from Ontario, Canada. Regions include Toronto (TOR), Brant (BRN), Lambton (LAM), Middlesex-London (MSL), Peel (PEE), Windsor-Essex (WEC), Haldimand-Norfolk (HDN); Lambton (LAM); Niagara (NIA); and Peterborough (PTC).

**Table S2.** Primer and probe sequences for real time TaqMan PCR for West Nile virus detection from mosquitoes. All primer and probe sequences were taken from Lanciotti et al. (2000) and ordered from Biosearch Technologies, (Novato, California USA). The 5’ reporter dye FAM was used and the 3’ quencher was TAMRA.

| **Primer/probe** | **5’ to 3’ sequence** |
| --- | --- |
| **WNV**  **WN3’NC-forward** | **CAGACCACGCTACGGCG** |
| **WN3’NC-reverse** | **CTAGGGCCGCGTGGG** |
| **WN3’NC-probe** | **TCTGCGGAGAGTGCAGTCTGCGAT** |
| **WNENV-forward** | **TCAGCGATCTCTCCACCAAAG** |
| **WNENV-reverse** | **GGGTCAGCACGTTTGTCATTG** |
| **WNENV-probe** | **TGCCCGACCATGGGAGAAGCTC** |

**Table S3.** Topological congruencies of phylosymbiosis between host phylogeny and microbiota dendrograms.

|  | **97% OTU clustering**  **Normalized Robinson-Foulds metric** | **99% OTU clustering**  **Normalized Robinson-Foulds metric** |
| --- | --- | --- |
| **Weighted Unifrac** | 0.75 (P=0.25) | 0.40 (P=0.0004) |
| **Unweighted Unifrac** | 1.0 | 0.60 (P=0.03637) |
| **Bray Curtis** | 1.0 | 0.40 (P=0.0004) |

**Table S4.** Statistical evaluation of pairwise differences between microbial communities of mosquito hosts. R^2^ values were calculated for the entire dataset of single isolates (top) and the dataset missing all Wolbachia OTUs (bottom).

|  | *Aedes vexans* complex | *Anopheles punctipennis* | *Anopheles quadrimaculatus* | *Coquilletidia perturbans* | *Culex pipiens/restuans* | *Culex salinarius* | *Ochlerotatus canadensis* | *Ochlerotatus japonicus* | *Ochlerotatus stimulans* | *Ochlerotatus triseriatus* | *Ochlerotatus trivitatus* |
| --- | --- | --- | --- | --- | --- | --- | --- | --- | --- | --- | --- |
| *Aedes vexans* complex | x |  |  |  |  |  |  |  |  |  |  |
| *Anopheles punctipennis* | **0.03432** | x |  |  |  |  |  |  |  |  |  |
| *Anopheles quadrimaculatus* | 0.02103 | **0.0603** | x |  |  |  |  |  |  |  |  |
| *Coquilletidia perturbans* | **0.13606** | **0.28281** | **0.22427** | x |  |  |  |  |  |  |  |
| *Culex pipiens/restuans* | **0.12319** | **0.1892** | **0.13677** | **0.2066** | x |  |  |  |  |  |  |
| *Culex salinarius* | **0.04878** | **0.19092** | **0.14632** | **0.16553** | 0.02191 | x |  |  |  |  |  |
| *Ochlerotatus canadensis* | 0.02039 | **0.10893** | 0.0811 | **0.17572** | 0.07313 | **0.19683** | x |  |  |  |  |
| *Ochlerotatus japonicus* | 0.02596 | 0.03126 | 0.04153 | **0.2547** | **0.16935** | **0.17399** | **0.09478** | x |  |  |  |
| *Ochlerotatus stimulans* | **0.0438** | **0.18047** | 0.13863 | **0.17614** | **0.09395** | 0.2509 | 0.14668 | **0.16122** | x |  |  |
| *Ochlerotatus triseriatus* | 0.02308 | 0.03381 | 0.06292 | **0.23942** | **0.11533** | 0.3146 | **0.18659** | 0.04181 | 0.29413 | x |  |
| *Ochlerotatus trivitatus* | 0.01377 | 0.07733 | 0.05899 | **0.2034** | **0.09767** | 0.27121 | 0.1295 | 0.06471 | 0.24217 | 0.14863 | x |
|  |  |  |  |  |  |  |  |  |  |  |  |
| *Aedes vexans* complex | x |  |  |  |  |  |  |  |  |  |  |
| *Anopheles punctipennis* | **0.03592** | x |  |  |  |  |  |  |  |  |  |
| *Anopheles quadrimaculatus* | 0.02156 | **0.06367** | x |  |  |  |  |  |  |  |  |
| *Coquilletidia perturbans* | 0.02751 | **0.07122** | **0.05821** | x |  |  |  |  |  |  |  |
| *Culex pipiens/restuans* | 0.0172 | **0.04792** | 0.02448 | 0.03109 | x |  |  |  |  |  |  |
| *Culex salinarius* | 0.01784 | 0.07555 | 0.05139 | 0.04006 | 0.02382 | x |  |  |  |  |  |
| *Ochlerotatus canadensis* | 0.02045 | **0.11427** | 0.08237 | 0.06943 | 0.02949 | 0.12745 | x |  |  |  |  |
| *Ochlerotatus japonicus* | 0.02584 | 0.03376 | 0.04133 | 0.05764 | 0.03463 | 0.06363 | **0.09481** | x |  |  |  |
| *Ochlerotatus stimulans* | **0.04521** | **0.18966** | 0.14189 | 0.10685 | **0.06159** | 0.20133 | 0.14829 | **0.16693** | x |  |  |
| *Ochlerotatus triseriatus* | 0.02291 | 0.03398 | 0.06112 | 0.06963 | 0.03465 | 0.14602 | **0.18655** | 0.04109 | 0.3027 | x |  |
| *Ochlerotatus trivitatus* | 0.01338 | 0.07761 | 0.05583 | 0.06166 | 0.0275 | 0.13184 | 0.12065 | 0.06643 | 0.24344 | 0.14705 | x |
| *Confidence: | **99%** | 95% |  |  |  |  |  |  |  |  |  |

**Table S5**. OTUs with significant seasonal trends in *Aedes vexans* complex and *Culex pipiens/restuans*. For seasonal OTUs and sequence reads based on the deblurred table without filtering for non-16S bacterial sequences, see Supplemental file: seasonal_OTUs.xlsx.

**Table S6**. Analysis of West Nile virus (WNV) prevalence in pooled samples (n=538 samples across three 3 years 2011-2013 from Toronto, Ontario) of *Culex pipiens/restuans*. WNV tested for correlation with mean air temperature, precipitation, and *Wolbachia* abundance in ambient conditions or conditions from prior sampling weeks. While *Wolbachia* was immediately reduced as temperature increased (Fig. 8A), WNV prevalence decreased 3-4 weeks later.

|  |  | WNV prevalence vs. | | | | |
| --- | --- | --- | --- | --- | --- | --- |
| Environmental conditions: |  | Ambient | 1 week prior | 2 weeks prior | 3 weeks prior | 4 weeks prior |
| Mean air temperature  (°C) N=44 | Pearson correlation | -0.072 | 0.121 | 0.267 | **0.425** | **0.448** |
|  | Significance (2-tailed) | 0.642 | 0.433 | 0.080 | **0.004** | **0.002** |
|  |  |  |  |  |  |  |
| Precipitation (mm) N=44 | Pearson correlation | 0.024 | 0.087 | 0.160 | 0.156 | 0.046 |
|  | Significance (2-tailed) | 0.878 | 0.573 | 0.300 | 0.313 | 0.765 |
|  |  |  |  |  |  |  |
| *Wolbachia* abundance | Pearson correlation | 0.076 | -0.075 | -0.298 | **-0.410** | -0.354 |
|  | Significance (2-tailed) | 0.665 | 0.674 | 0.092 | **0.020** | 0.051 |
|  | N | 35 | 34 | 33 | **32** | 31 |

**Supplemental Figures**

**Figure S1.** Number of mosquitoes captured each week. Three years of data combined from 2011-2013.

**Figure S2.** Relative abundances of bacterial taxa in different host species, based on pooled samples, and their relationship to the host phylogeny. Bold printed taxa were congruently found in abundances above 2% for the single samples (Fig. 3).

**Figure S3.** Maximum likelihood based phylogenetic relationships among all *Wolbachia* OTUs found in the complete dataset. The numbers at the nodes stand for bootstrap values. The tip numbers are the OTU designations. The gray values represent relative abundance in each species (calculated as a percentage of mean read numbers, for each of the OTUs per species, out of the average total read number for a pooled sample, i.e. 82,124). Our method of assigning OTU taxonomy with RDP suggested 17 *Wolbachia* OTUs. However, this phylogenetic analyses determined only 13 of these to belong within the genus *Wolbachia*.


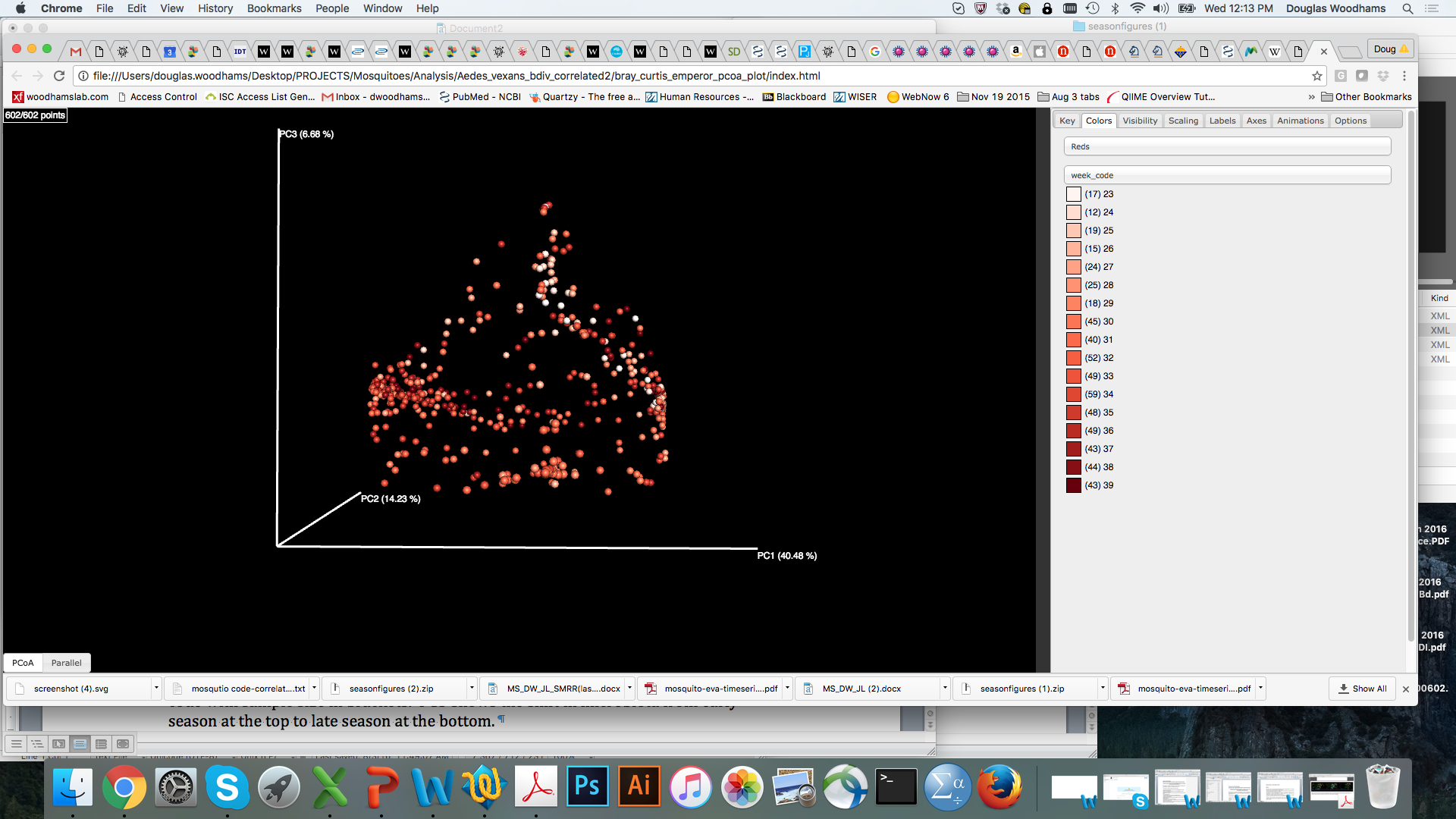

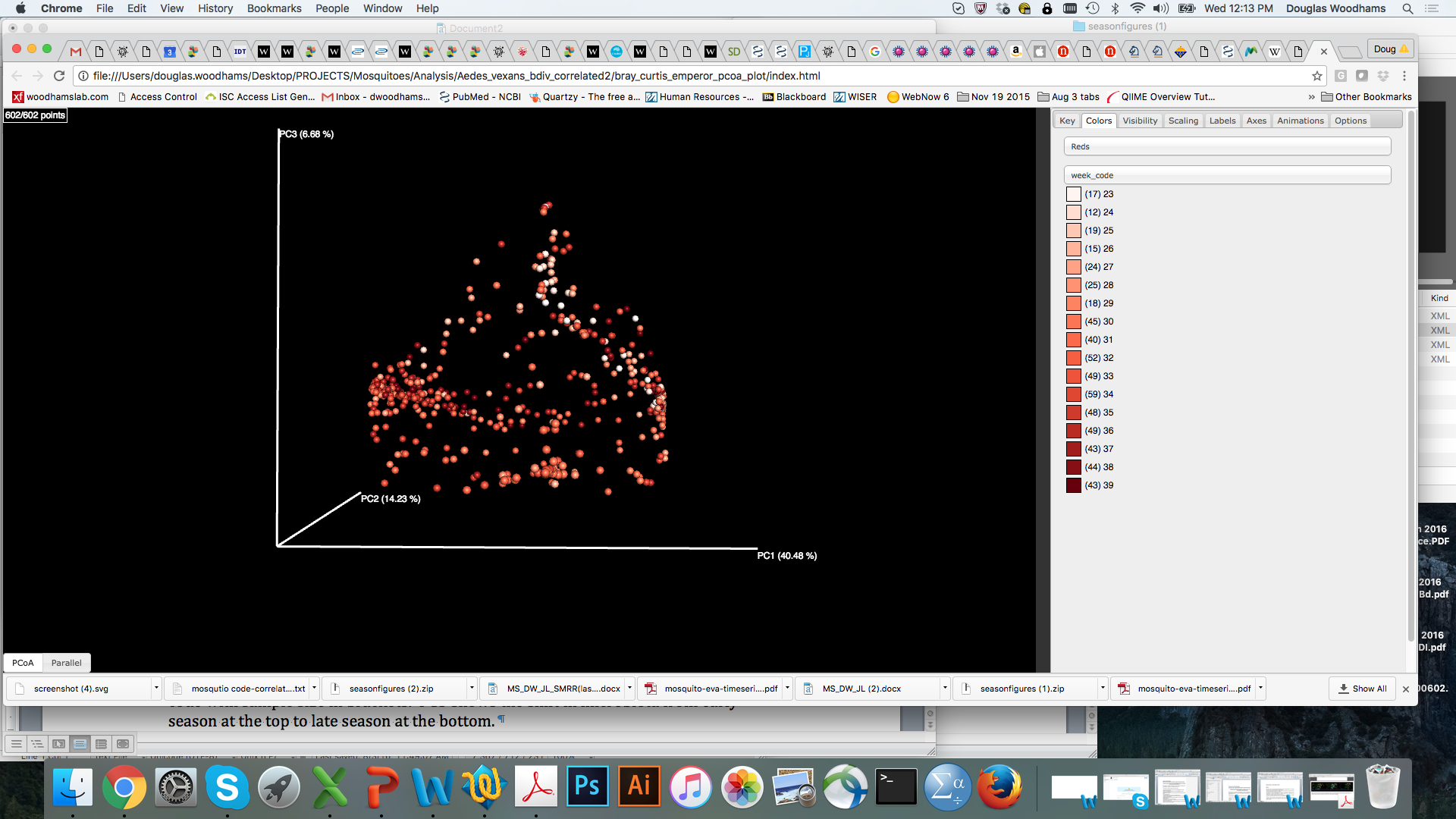


**Figure S4.** Beta-diversity principal coordinates plot of microbiota from *Aedes vexans* complex mosquitoes sampled between 2011-2013. Legend indicates epidemiological week with sample size in brackets. Early and late season clustering is suggestive of a shift in mid-summer (lower left cluster) and then a return to previous microbial communities.


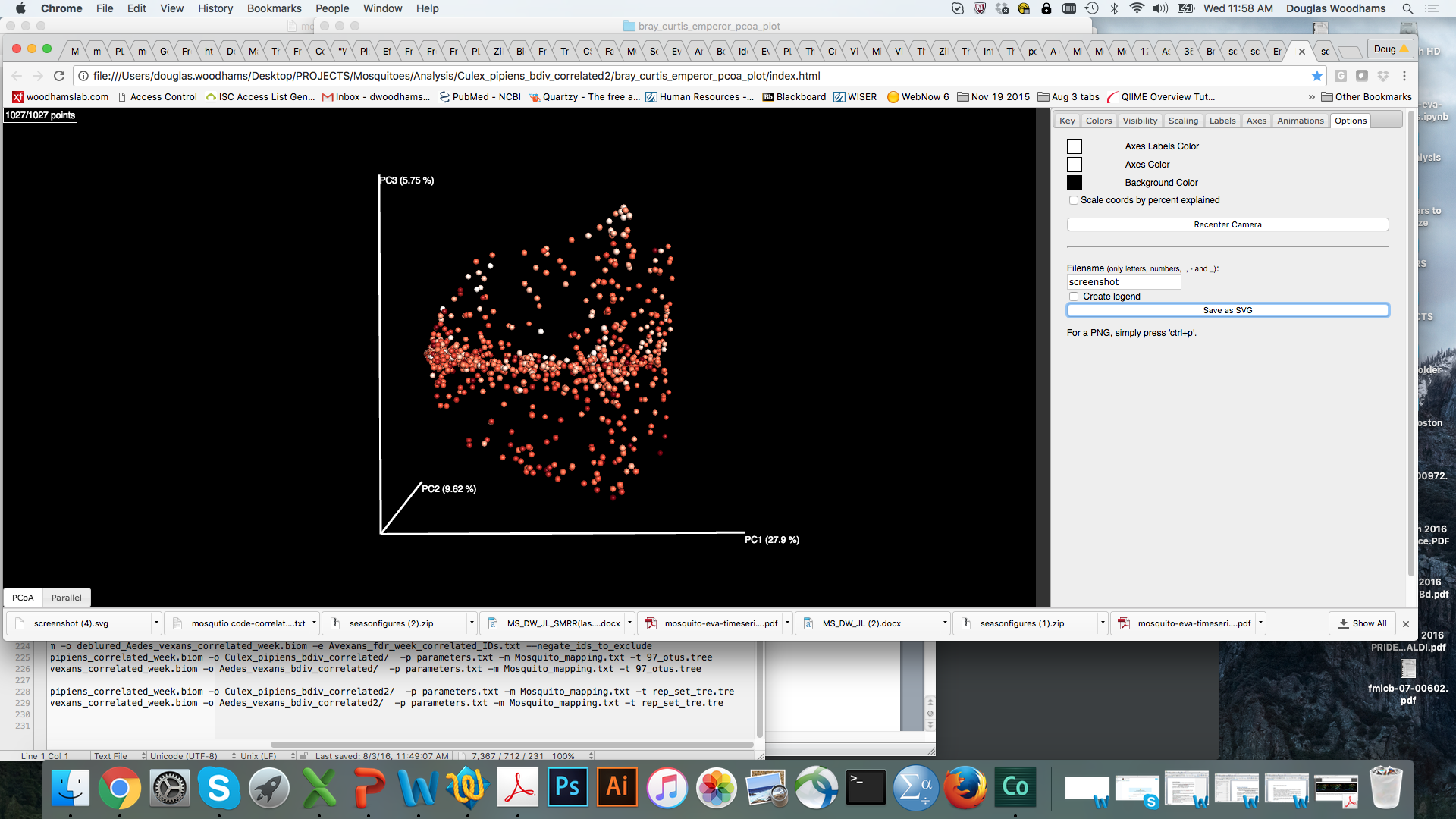

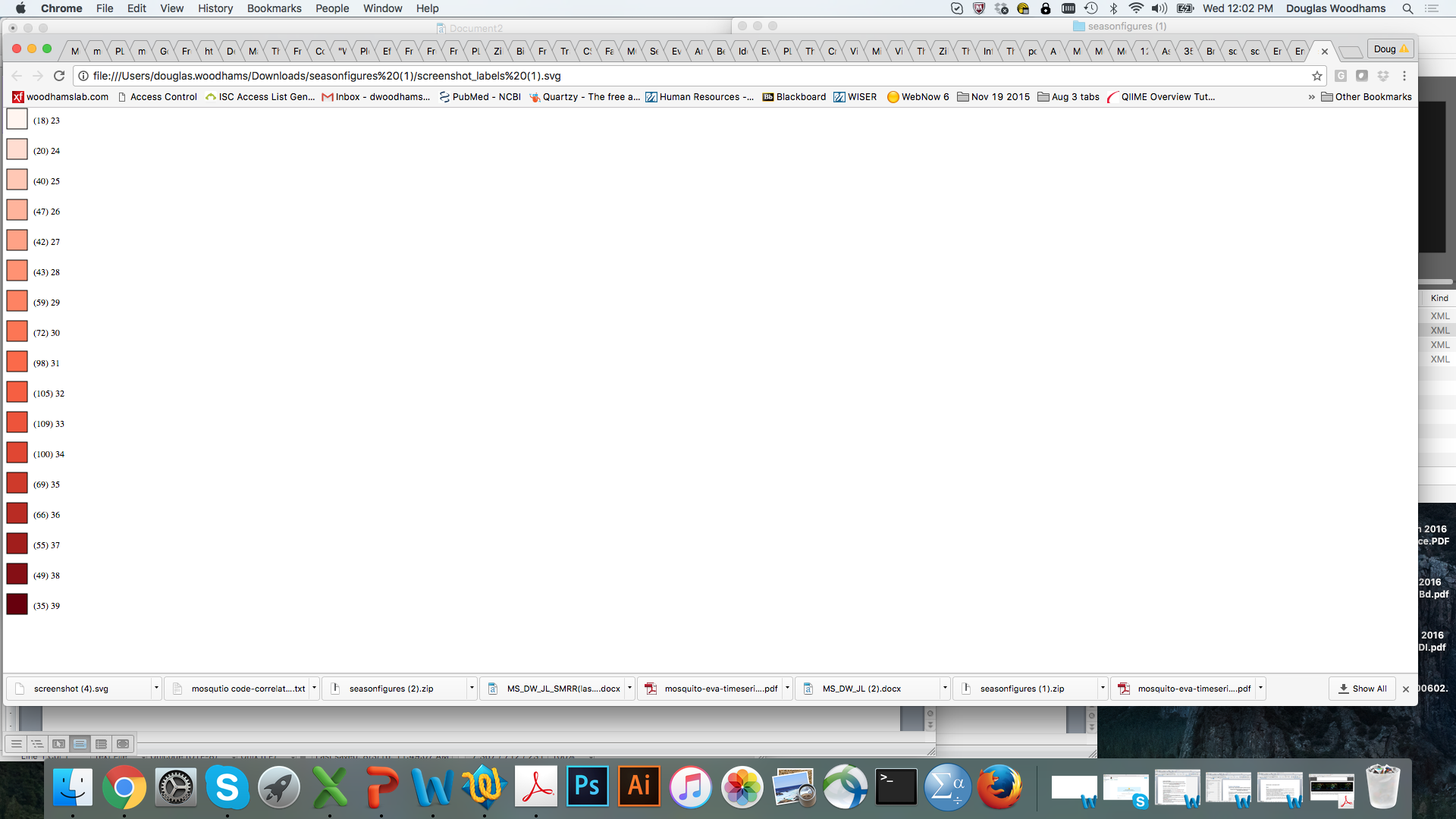


**Figure S5.** Beta-diversity principal coordinates plot of microbiota from *Culex pipiens* complex mosquitoes sampled between 2011-2013. Legend indicates epidemiological week with sample size in brackets. PC3 shows the shift in microbiota from early season at the top to late season at the bottom

**Figure S6.** Heatmap (dark blue = low abundance, red = high abundance) of OTUs from *Aedes vexans* complex pooled samples shifting in relative abundance through sampling week (three years combined 2011-2013).

**Figure S7.** Heatmap (dark blue = low abundance, red = high abundance) of OTUs from *Culex pipiens/restuans* complex pooled samples shifting in relative abundance through sampling week (three years combined 2011-2013).

**Supplemental References**

Darsie RF, Ward RA (2005). Identification and geographical distribution of the mosquitoes of North America, North of Mexico. University Press of Florida.

Thielman AC, Hunter FF (2007). A photographic key to adult female mosquito species of Canada (Diptera: Culicidae). *Canadian Journal of Arthropod Identification*, 4.

Lanciotti RS, Kerst AJ, Nasci RS, Godsey MS, Mitchell CJ, Savage HM, Komar N, Panella NA, Allen BC (2000). Rapid detection of West Nile virus from human clinical specimens, field collected mosquitoes and avian samples by a TaqMan®RT-PCR assay. *J. Clin. Microbiol*. **38**: 4066-4071.
